# Reprogramming of macrophages employing gene regulatory and metabolic network models

**DOI:** 10.1371/journal.pcbi.1007657

**Published:** 2020-02-25

**Authors:** Franziska Hörhold, David Eisel, Marcus Oswald, Amol Kolte, Daniela Röll, Wolfram Osen, Stefan B. Eichmüller, Rainer König

**Affiliations:** 1 Center for Sepsis Control and Care, University Hospital, Jena, Germany; 2 Research Group GMP & T Cell Therapy, German Cancer Research Center (DKFZ), Heidelberg, Germany; 3 Biopharmaceutical New Technologies (BioNTech) Corporation, Mainz, Germany; US Army Medical Research and Materiel Command, UNITED STATES

## Abstract

Upon exposure to different stimuli, resting macrophages undergo classical or alternative polarization into distinct phenotypes that can cause fatal dysfunction in a large range of diseases, such as systemic infection leading to sepsis or the generation of an immunosuppressive tumor microenvironment. Investigating gene regulatory and metabolic networks, we observed two metabolic switches during polarization. Most prominently, anaerobic glycolysis was utilized by M1-polarized macrophages, while the biosynthesis of inosine monophosphate was upregulated in M2-polarized macrophages. Moreover, we observed a switch in the urea cycle. Gene regulatory network models revealed E2F1, MYC, PPARγ and STAT6 to be the major players in the distinct signatures of these polarization events. Employing functional assays targeting these regulators, we observed the repolarization of M2-like cells into M1-like cells, as evidenced by their specific gene expression signatures and cytokine secretion profiles. The predicted regulators are essential to maintaining the M2-like phenotype and function and thus represent potential targets for the therapeutic reprogramming of immunosuppressive M2-like macrophages.

## Author summary

The innate immune system is the first defense line to infection and macrophages are its central players. Macrophages polarize into their resistant state when sensing an invading pathogen. In turn, they can also polarize into a resilient state and provide compounds for anabolism to e.g. promote wound healing processes. In several diseases ranging from cancer to auto immune diseases and sepsis, the disturbed polarization of macrophages is critically involved in their patho-mechanism. We investigated transcription profiles of polarized macrophages to identify central regulators aiming to reprogram detrimental polarization when targeted. Indeed, we came up with a model containing four transcription factors that would reprogram macrophages based on an assembled gene signature representing the distinct regulation of these polarized macrophages. Experimentally silencing these transcription factors validated the predictions and showed that we can shift macrophages polarized from their resilient state toward the resistant state. Inhibiting these regulators in specific macrophages may enable reactivating them into resistance which may have a tumor suppressive effect of tumor associated macrophages or reactivating them during the immunosuppressive phase after sepsis.

## Introduction

Innate immunity serves as a first-line defense against pathogens and includes natural killer (NK) cells as well as phagocytic cells such as dendritic cells, neutrophils and macrophages [1]. Thus, macrophages are functionally involved in the control of various diseases such as cancer, diabetes, sepsis and chronic immunological disorders [2–5]. Macrophages show marked plasticity. Depending on the environmental stimuli they receive, macrophages polarize into myeloid cell types with distinct phenotypes and functions. Extensive research has led to a better understanding of the molecular mechanisms that control macrophage polarization [6–8]. IL-10 signaling has been shown to play a critical role in controlling inflammatory responses by modulating cellular metabolism in activated macrophages [7]. To simplify macrophage biology, the functional spectrum of macrophages is represented by the two extremes, i.e., classically activated M1-like macrophages and alternatively activated M2-like macrophages (subsequently denoted as M1-like and M2-like macrophages). M1-like macrophages, which are activated through IFN-γ as well as other factors, contribute to the inflammatory immune response by producing proinflammatory mediators such as TNF-α, IL-6 and nitric oxide. Furthermore, they eliminate invading pathogens by phagocytosis [9,10]. In contrast, M2-like macrophages, which can be activated by IL-4, show immunosuppressive functions and promote wound healing [11].

It is known that tumor cells can reprogram proinflammatory immune cells, particularly M1-like macrophages, into anti-inflammatory immune cells, including M2-like macrophages, to induce local immune suppression [12,13]. Specifically, tumor-associated macrophages (TAMs) are recruited by the tumor through the secretion of chemoattractants. TAMs are involved in tumor progression and are characterized by high secretion of growth factors and anti-inflammatory cytokines, promoting angiogenesis, cancer growth and tissue infiltration [14]. Thus, immunotherapies aiming to polarize TAMs into M1-like macrophages have been suggested as a therapeutic approach against cancer [15].

In addition, macrophages play a central role during sepsis, which is a life-threatening condition resulting from organ dysfunction as a consequence of systemic infection. Organ dysfunction is caused by a hyperreactive immune response against an infection in the blood. Notably, the initial proinflammatory response is followed by an anti-inflammatory immune response, increasing patient susceptibility to severe secondary infections during sepsis. Hence, therapies that enhance the immune response against the primary infection may increase the survival of patients suffering from sepsis [16].

Interestingly, macrophages analyzed in murine sepsis models show marked functional plasticity. Thus, peritoneal macrophages derived from mice with induced polymicrobial sepsis were shown to be unresponsive to endotoxin (lipopolysaccharide, LPS) stimulation and to secrete reduced amounts of TNF-α, IL-6, and IL-1β, while IL-10 levels were increased, thus reflecting an immunosuppressive M2-like phenotype. At the same time, the chemokine expression profile of these macrophages suggested an M1-like phenotype, as the gene expression of CCL2 (MCP1) was increased, whereas the expression of CCL17, CCL22, and CCL24 was reduced [5]. Another study showed that the reprogramming of macrophages from an M1-like phenotype to an M2-like phenotype can be induced by IL-4, and conversely, a phenotypic switch from the M2-like phenotype to the M1-like phenotype was observed upon the addition of M1-polarizing stimuli [17]. Recently, we showed that M2-like macrophages can be reprogrammed to the M1-like phenotype upon an antigen-specific interaction with CD4^+^ T cells [18]. Hence, the repolarization of macrophages is feasible; however, the underlying regulatory mechanisms are still elusive. In response to the microenvironment, resting macrophages not only polarize into distinct phenotypes but also develop specific metabolic demands. A recent study reported two breakpoints in the citrate cycle of M1-like macrophages supporting the production of the anti-inflammatory metabolite itaconate [10]. Furthermore, bone marrow-derived macrophages from IL-10 knockout mice showed higher glucose uptake and upregulated glycolysis and decreased mitochondrial oxygen consumption, suggesting that IL-10 promotes oxidative phosphorylation [7].

To better understand the processes regulating M1/M2 polarization, we investigated the gene regulation of metabolism and identified regulators that are essential in M2-like macrophages providing a possible starting point to shift M2-like macrophages into M1-like macrophages.

## Results

### Differentially expressed gene sets in M1- and M2-like macrophages

**Fig 1:**
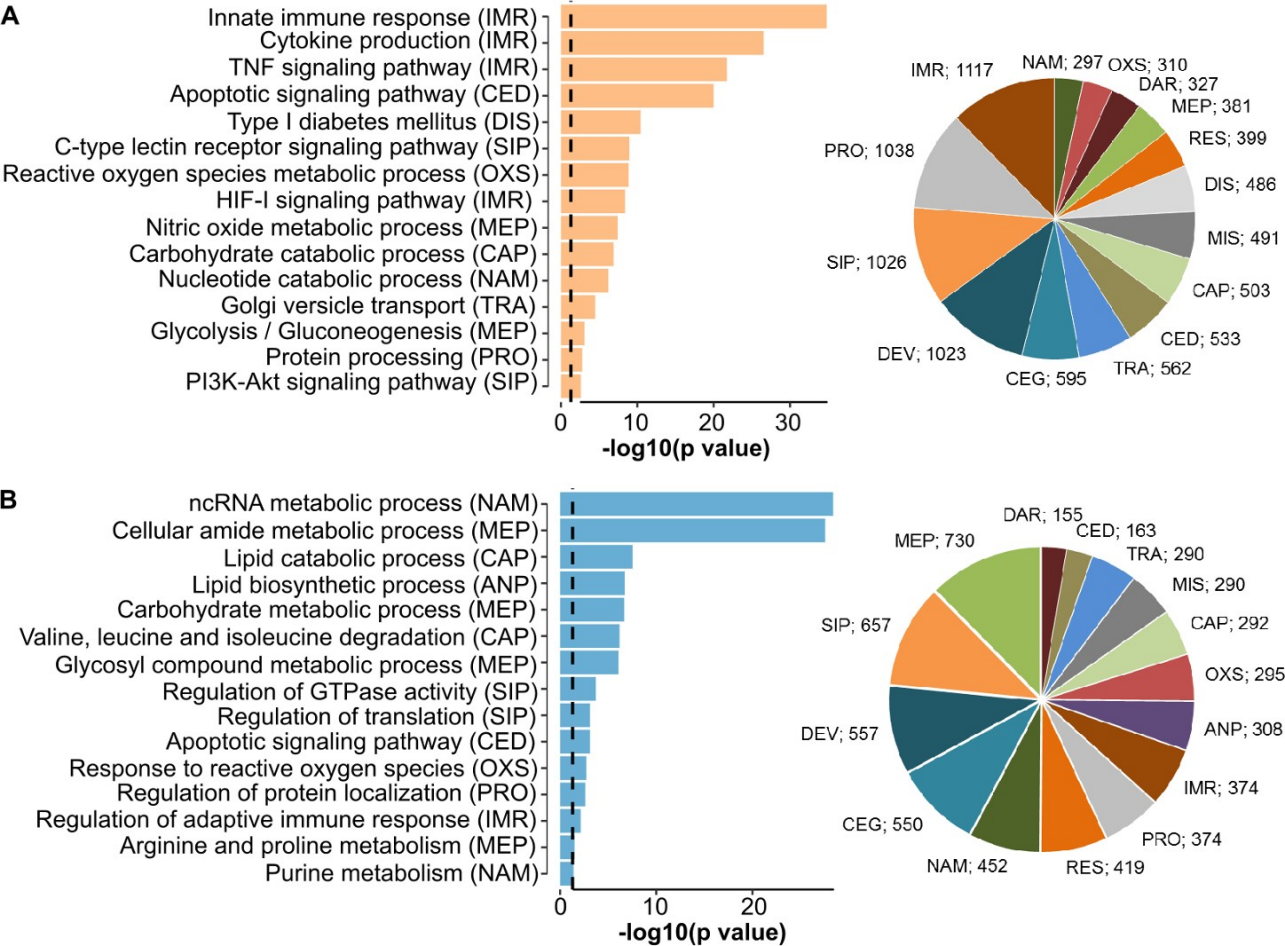
Shown is a representative selection of gene sets derived from gene set enrichment analysis of RNA-seq data (S1 Table) with upregulated expression in macrophages polarized to the M1-like phenotype (A) or M2-like phenotype (B) in respect to the other polarization. Genes with upregulated expression in M1-like macrophages were significantly enriched in the categories immune response (IMR, P = 1.67E-48), cell death (P = 4.55E-31), signaling processes (P = 2.72E-07) and specific metabolic processes such as nitric oxide synthesis and glycolysis, while genes with upregulated expression in M2-polarized macrophages were enriched in anabolism (P = 1.22E-96), metabolic processes (P = 8.44E-30) and nucleotide metabolism (P = 7.31E-12). Enrichment tests were performed using g-Profiler.

Mice were injected intraperitoneally with thioglycolate containing medium to get non-polarized macrophages in the peritoneum (Peritoneal exudate cells, PEC cells). Polarization into M(LPS+IFNγ) and M(IL4) macrophages was induced by LPS + IFNγ and IL4, respectively obtaining M1-like and M2-like polarized macrophages. In the following, we refer to M1-like and M2-like macrophages for these M(LPS+IFNγ) and M(IL4) macrophages. We compared the transcription profiles of M1-like and M2-like polarized macrophages (n = 3 replicates), selected differentially expressed genes, and performed gene set enrichment analysis. S1 Table lists all significantly differentially expressed genes. Significantly enriched gene sets were grouped into 15 major categories of cellular processes, and statistical tests were performed to identify the categories with significantly different numbers of genes with upregulated expression (Fig 1). As expected, we found significantly more genes with upregulated expression for immune response processes in the M1-like macrophages than in the M2-like macrophages (P = 1.67E-48, g:Profiler statistic, 1,117 genes in the M1-like macrophages, 374 genes in the M2-like macrophages). In particular, the M1-like macrophages showed a strong upregulation of the expression of immune mediators (Il-6, Tnf-α, and Stat1; see S1 Fig), which stimulate the proinflammatory response. Furthermore, in the M1-like macrophages, we observed more genes with upregulated expression for cell death (P = 4.55E-31, g:Profiler statistic), cellular signaling (P = 2.72E-07, g:Profiler statistic), such as TNFα signaling and HIF-1α signaling, and protein transport and modification (P = 1.31E-40, g:Profiler statistic), reflecting the extensive upregulation of cellular processes in response to extrinsic stressors. The Kyoto Encyclopedia of Genes and Genomes (KEGG) provides gene sets associated with diseases. Only for the M1-like macrophages, we found that the disease-related gene sets with upregulated expression mainly comprised diseases caused by infection (n = 359), autoimmune diseases (n = 60) and cancer (n = 242) (S2 Table). Comparing the two types of macrophages indicated that both the M1- and M2-like macrophages showed upregulation of cell cycle and repair mechanisms. Cell cycle mechanisms in the context of M2-like macrophages have been described before [19]. However, we also observed upregulation of gene sets of cell cycle categories in the M1-like macrophages (e.g., cell cycle) and found inflammatory genes such as Il1α, Il1β, Il12α and Il12β to be associated with the Gene Ontology definitions for cell cycle processes, possibly explaining this observation. For the M2-like macrophages, most of the gene sets with upregulated expression belonged to metabolic processes, as described in the next section. Fig 1 displays the categories and a representative selection of the gene sets upregulated in the M1-like and M2-like macrophages. All identified gene sets are listed in S2 Table in the Supplementary Material.

In summary, differential gene expression analysis showed that M1-like macrophages activated inflammatory processes such as inflammation, cell death, proinflammatory cytokine expression, and TNF signaling, while M2-like macrophages upregulated metabolic and cellular maintenance processes.

### Distinct regulation of metabolism in M1- and M2-like macrophages: glycolysis and catabolism *versus* anabolism

In M1-like macrophages, glycolysis, comprising 22 genes with upregulated expression such as hexokinases 1–3, Gapdh and Ldha, was upregulated (P = 8.27E-04 using g:Profiler). Notably, the entire biochemical pathway from glucose to lactate production was upregulated. In addition, processes affecting the catabolism of nucleotides, cellular macromolecules and carbohydrates were upregulated. In contrast, M2-like macrophages showed upregulation of anabolic processes such as the biosynthesis of amino acids and nucleic acids. Enhanced fatty acid metabolism and expression of mitochondrial genes reflected upregulated oxidative phosphorylation (Fig 1, S2 Table). The biosynthesis of inosine monophosphate (IMP) is the initial and rate-limiting step in purine biosynthesis. We observed the entire pathway for IMP biosynthesis to be upregulated in the M2-like macrophages (n = 7 out of 7 genes).

Thus, M1-like macrophages upregulated biochemical catabolic processes and glycolysis, in particular all genes coding for the Warburg effect, which was also reflected by upregulated Hif1α signaling, whereas the M2-like macrophages showed upregulated nucleotide synthesis and oxidative phosphorylation, hinting at a distinctively different regulation of metabolism. These results initiated our interest in modeling the metabolic fluxes and their regulation described in the next sections.

### Constraint-based modeling suggests switches in metabolic fluxes

**Fig 2:**
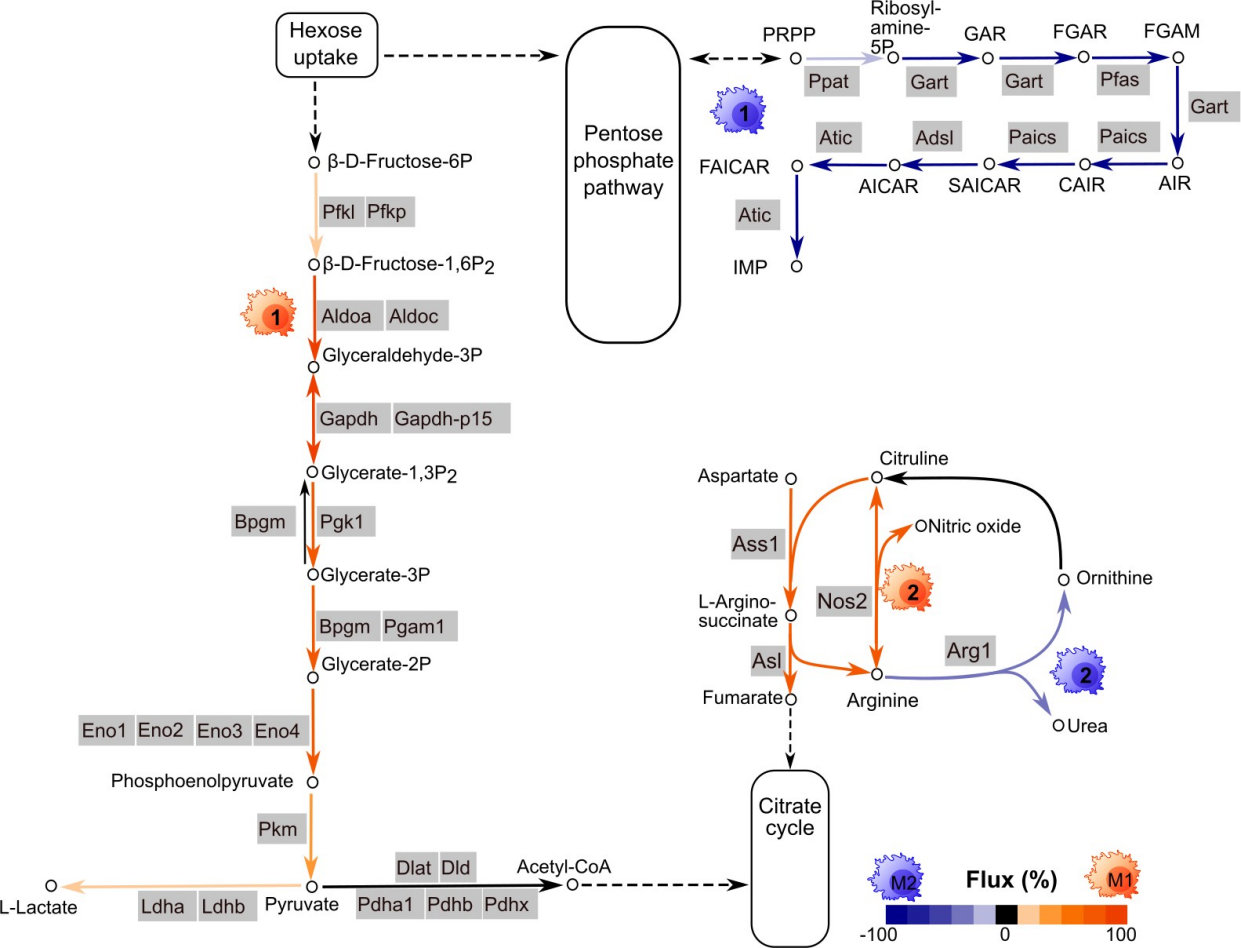
The metabolic fluxes of different macrophage phenotypes, M0, M1-like and M2-like, were predicted by integrating the gene expression of enzymes into a metabolic network model. The model revealed differential fluxes mainly involved in glycolysis, inosine monophosphate (IMP) synthesis, the urea cycle and lactate production. Orange arrows indicate higher metabolic fluxes in M1-like macrophages, and blue arrows indicate higher fluxes in M2-like macrophages. Induced glycolysis was observed in M1-polarized macrophages, while IMP synthesis was high in M2-like macrophages. The quantitative values are represented by the respective color intensity ranging from 0% (no differential flux between M1-like and M2-like macrophages) and 100% (the predicted flux was observed only in the according macrophage type, and no predicted flux was observed in the other polarized macrophages).

We studied the difference in the regulation of metabolism observed in a mechanistic way employing constraint-based modeling and the murine macrophage model described in Bordbar *et al* (2012)[20]. In our approach, we estimated the metabolic flux with a linear model that assumed the metabolic flux was linearly dependent on the expression of the enzyme encoded by the respective gene and was constrained by the interconnected stoichiometry of the substrates and products in the metabolic network. These constraints are based on the well-established concept of flux balance analysis (see, e.g., Orth *et al* (2010)[21], for details, see Materials and Methods). Our models supported the above-described duality between glycolysis and nucleotide biosynthesis and revealed a switch-like behavior in the metabolic fluxes (Fig 2). The predicted metabolic flux of reactions involved in glycolysis and lactate production was distinctively higher in M1-like macrophages than in M2-like macrophages (S3 Table). In contrast, in M2-like macrophages, there was a higher flux in the biosynthesis of IMP, confirming the above-suggested switch between glycolysis and IMP biosynthesis. Moreover, in our model, there was a higher flux for nitric oxide synthase in M1-like macrophages, while there was a higher flux for arginase in M2-like macrophages. This finding suggests a second switch between nitrogen being either metabolized into nitric oxide as a proinflammatory signaling molecule (in M1-like macrophages) or degraded into urea by arginase (in M2-like macrophages). Indeed, nitric oxide synthase and arginase have been described as valid markers of murine M1- and M2-like macrophages, respectively [10]. Notably, we observed a similar metabolic flux profile for M0 and M2-like macrophages. The metabolic flux for IMP synthesis was also higher in M0 macrophages than in M1-like macrophages. In contrast, there was no metabolic flux through the urea cycle and no synthesis of lactate in M0 macrophages. The estimated fluxes for M0 macrophages are also provided in S3 Table in the Supplementary Material.

In summary, flux balance analysis elucidated metabolic switches between M1-like and M2-like polarization, most prominently between glycolysis and purine biosynthesis and between nitric oxide and urea production in the urea cycle.

### Compiling a literature-based gene expression signature associated with M1- and M2-polarized macrophages

Macrophages are classified according to their origin and functional features. Various macrophage subsets have been reported. The activation signals of Th2 responses, such as the secretion of IL-4 or IL-13, trigger M2-like polarization in macrophages, while M1-like macrophages are induced by exposure to LPS and IFN-γ [10]. Previous studies have demonstrated distinct transcriptional signatures for M1- and M2-like-polarized macrophages, providing a reasonable means for the experimental validation of these distinct macrophage subsets. Hence, based on the studies listed in S4 Table, we compiled a list of 36 genes for an initial transcriptional gene signature distinguishing M1- and M2-like polarization. The signature comprises surface markers (i.e., Cd80, Cd86), cytokines, chemokines, metabolic enzymes (Arg1, Nos2 or iNos) and other M1/M2 markers, such as Chil3 (Ym1), Retnla (Fizz1) and Vegfa/b. M1-like macrophages express high levels of proinflammatory mediators such as IL-6 and TNF-α. In contrast, M2-like macrophage activation leads to the upregulation of Il-10 expression, while the expression of Il-12 is reduced [8]. The gene expression profile of our M1-like- and M2-like-polarized macrophages was in line with the expression reported in the literature (n = 33 agreed, n = 3 disagreed, S4 Table). As expected, compared to the M2-like macrophages, the M1-like macrophages upregulated the expression of proinflammatory cytokines and chemokines such as Il6, Il12, Tnfα, Ccl3 and Ccl4. In contrast, Tgfb2 and tissue repair genes such as Chil3 and Retnla exhibited upregulated expression in the M2-like macrophages and were associated with M2-like polarization. In the rest of the manuscript, the described list is denoted as the literature signature (S2 Fig).

### In silico reprogramming of activated macrophages reveals key regulators for the metabolic switch

**Fig 3:**
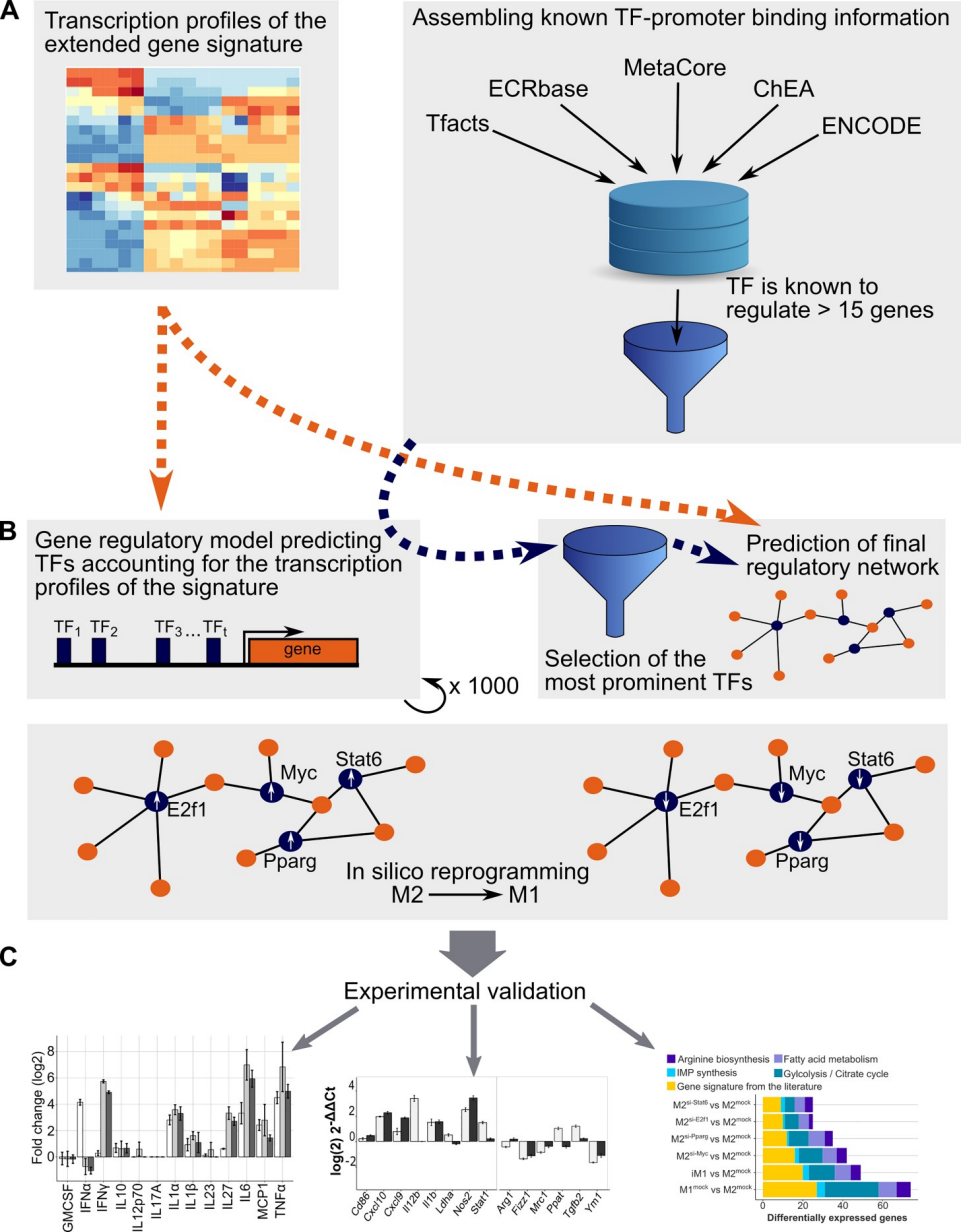
The analysis pipeline to obtain the gene regulatory model. (A) Gene expression profiles and evidence-based interactions for all transcription factors were collected. (B) After a pre-filtering step, the activity of each transcription factor was determined, and regulatory network models were generated. The transcription factors that were most often selected by the models across all target genes were incorporated into the extended gene signature, a parsimony model constructed, and *in silico* reprogramming was performed to convert the activities of these transcription factors into M1-like activities. (C) The predicted transcription factors were experimentally validated by investigating the cytokine secretion and transcription profiles of the reprogrammed M2-like macrophages after knocking down the expression of the predicted transcription factors.

**Fig 4:**
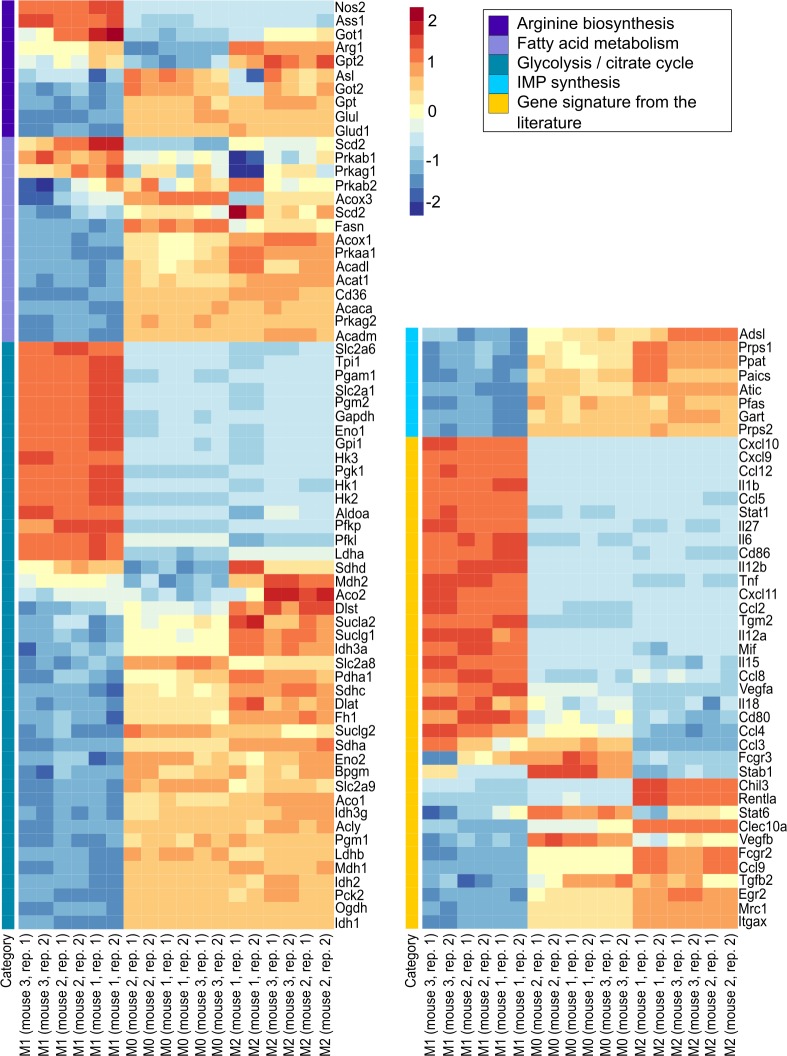
The relative expression (z-scores) of the extended gene signature comprising genes from arginine biosynthesis, fatty acid metabolism, glycolysis, the citrate cycle, IMP biosynthesis and the literature gene signature is shown. Red: upregulation, blue: downregulation, the columns represent biological (mouse 1, 2, 3) and technical (repl. 1, 2) replicates of M1-like, M0 and M2-like macrophages.

Gene set enrichment analysis and mechanistic modeling revealed distinct regulation of central energy metabolism, anabolism and catabolism in M1- and M2-like macrophages. Next, we aimed to identify the transcriptional regulators of this regulatory switch. Our detailed workflow was as follows (Fig 3): first, we selected 76 metabolic genes of enriched pathways of energy metabolism including also genes of the citrate cycle and the pentose phosphate pathway. We only considered genes with differential expression among the selected biochemical pathways including glycolysis, citrate cycle, the pentose phosphate pathway, fatty acid metabolism, IMP synthesis and arginine biosynthesis. To find the regulators that are also responsible for the differential expression of M1- and M2-like associated genes described in the literature, we added the 36 genes of the literature signature described in the previous section. This led to a list of 112 differentially expressed genes subsequently denoted as the *extended gene signature* (the list is given in Fig 4). We predicted the potential regulators of these genes by estimating their gene expression profiles employing linear regression models. For each of these target genes, a model was set up. Each model contained an optimization parameter and the activity of each transcription factor (TFs) for which binding information was found in ChIP databases. The objective was to minimize the difference between the predicted and measured gene expression [22]. The models were constrained to select the same sets of regulators for several target genes employing Mixed Integer Linear Programming (constraint-based modeling). After employing a prioritization strategy, the nine top-ranking transcription factors were selected as regulators of all the genes in our signature. For reprogramming M2-like macrophages into M1-like macrophages *in silico*, we replaced the activities of the predicted regulators in M2-like macrophages with their activities in M1-like macrophages.

Following this strategy we determined five transcription factors, CTCF, E2F1, MYC, PPARγ, and STAT6, predicted to enable the reprogramming of M2-like macrophages into M1-like macrophages when silenced. S3 Fig shows the gene regulatory model. Interestingly, the activities of all five transcription factors were higher in M2-like macrophages than in M1-like macrophages. The majority of the genes were regulated by MYC followed by E2F1, CTCF, PPARγ and STAT6 (MYC: 72, E2F1: 57, CTCF: 28, PPARγ: 48, and STAT6: 14 genes). E2F1 and MYC were predicted to regulate a large cluster of metabolic genes, whereas the genes in the literature signature were predicted to be regulated mainly by PPARγ and STAT6. In our gene regulatory model, 85% of the observed genes switched their expression from an M2-like state into an M1-like state (S4 Fig). We also analyzed the performance of smaller models consisting of fewer regulators. Overall, the agreement with an M1-like expression pattern of the different combinations of the predicted transcription factors decreased when decreasing the number of regulators in the models. Employing the best combinations of four transcription factors, more than 78% and 76% of genes were in accordance with the extended gene signature of M1-like macrophages. These combinations were CTCF, E2F1, MYC, and PPARγ and E2F1, MYC, PPARγ, and STAT6, respectively. The latter combination yielded better predictions for the genes of the literature signature and, hence, was used for subsequent investigations. S2 Fig shows the corresponding gene regulatory model.

In summary, we identified short lists of transcription factors, most prominently CTCF, E2F1, MYC, PPARγ and STAT6, predicted to enable the reprogramming of M2-like macrophages into M1-like macrophages when their expression was knocked down.

### M2-polarized macrophages shift to the inflammatory phenotype upon knocking down the expression of the predicted regulators *in vitro*

In the next step, the predicted transcription factors were functionally validated. All five transcription factors were predicted to maintain the M2-like phenotype. According to our modeling results described in the previous section, we hypothesized that knocking down the expression of Ctcf, E2f1, Myc, Pparγ and Stat6 in M2-like macrophages should induce their repolarization into proinflammatory M1-like macrophages. Therefore, we investigated the changes in polarization upon transcription factor expression knockdown by measuring gene expression and cytokine secretion in M2-like macrophages after transfection with siRNA pools targeting the four transcription factors E2f1, Myc, Pparγ and Stat6. Knockdown efficiency was tested for each of these TFs at 24h, 48h and 72h after siRNA transduction showing for all except one condition and TF (E2F1, 48h) expression reduction by at least 50% (S6 Fig).

**Fig 5:**
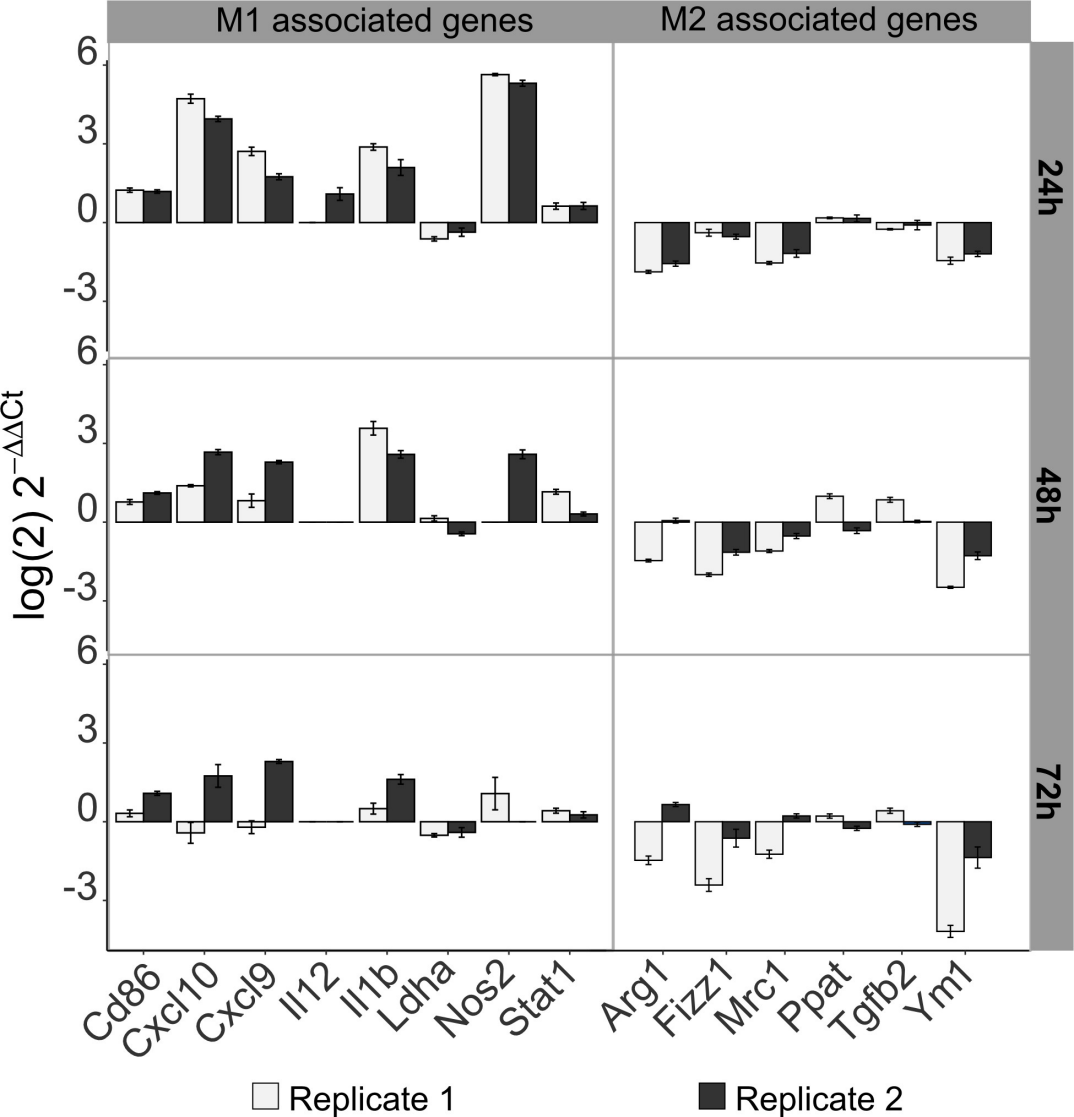
Transcriptional changes in M1-like- and M2-like-associated genes after knocking down the expression of four selected TFs were experimentally determined by quantitative PCR. The log2-fold changes in the gene expression of 15 genes from the literature signature in iM1 macrophages (M2-like after knockdown of E2f1, Myc, Pparγ, Stat6) relative to that in the (mock treated) M2-like macrophages are shown. Samples were extracted 24 h, 48 h and 72 h after siRNA treatment. Error bars are based on the standard error of technical replicates. Genes were considered successfully reprogrammed if a switch towards an M1-like phenotype was observed in both biological replicates (replicates 1 and 2 in gray and black, respectively).

As a first validation, we performed qPCR analysis of selected genes of the literature signature at three time points after transfection. The highest impact of the knockdown on the gene expression of M1-like and M2-like associated genes was observed at 24 h and 48 h after transfection with the combined siRNA pool (Fig 5, S5 Table). Ten and eight out of fourteen M1- and M2-associated genes, respectively, switched their gene expression as expected at 24 h and 48 h after transfection. Nos2, an important M1 marker, exhibited highly upregulated expression at 24 h and 48 h after knockdown treatment, while the expression of the M2 marker Arg1 was decreased 24 h and 48 h after knockdown treatment. Furthermore, the observed ratio of the expression of M1/M2 associated genes in iM1-like and M2-like macrophages was in line with the observed ratio of expression in M1 and (mock treated) M2-like macrophages, the same eleven out of 14 genes showed the same trend in differential expression (see S7 Fig). Notably, if these TFs were silenced in M0 macrophages, i.e. without IL-4 stimulation, we observed a similar behaviour (S9 Fig), suggesting that these TFs prevent M1 polarization regardless of IL-4 stimulation. Knocking down the expression of all five predicted transcription factors (E2f1, Myc, Pparγ, Stat6, and Ctcf) resulted in a very similar shift towards the M1-like phenotype (S5 Fig). These findings suggested that knocking down Ctcf expression is not necessarily required for successful M1-like repolarization. Thus, the Ctcf-targeting siRNA pool was excluded in subsequent experiments focusing on the four selected transcription factors, E2f1, Myc, Pparγ and Stat6. M2-like macrophages treated with the siRNA pool targeting these four transcription factors are subsequently denoted as induced M1-like (iM1) macrophages.

**Fig 6:**
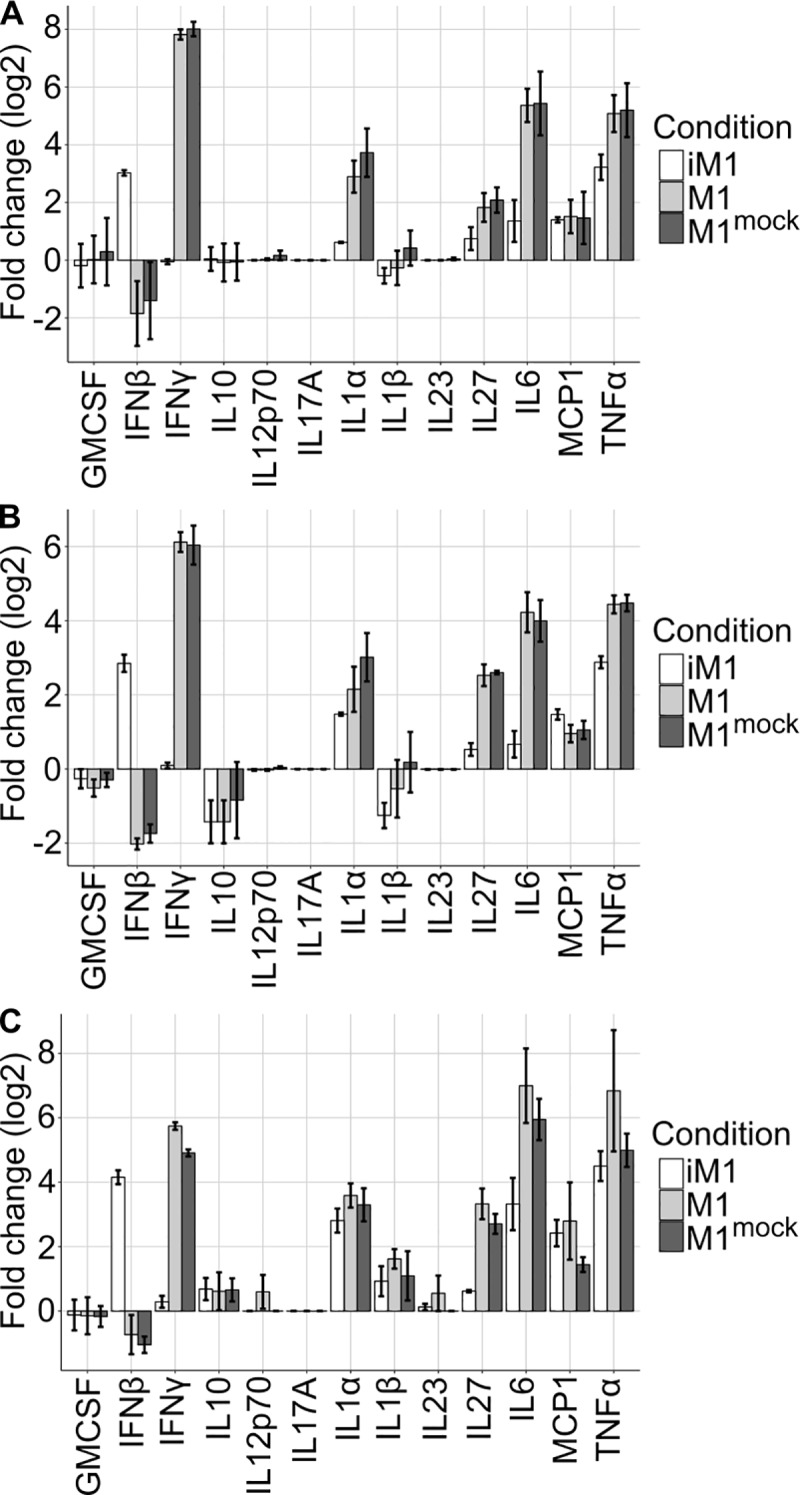
The quantification of cytokines released by M2-polarized macrophages upon transfection with siRNA pools was performed by multiplex analysis. Enhanced secretion of IFN-β, IL-1α, IL-6, IL-27, MCP1 and TNF-α but not IFN-γ was detected at 24 h (A), 48 h (B) and 72 h (C) after transfection in M2-like macrophages treated with the combined siRNA pool targeting E2f1, Myc, Pparγ and Stat6 (iM1) compared to (mock treated) M2-like macrophages.

Thirteen cytokines secreted by M2-polarized macrophages after transfection of the siRNA pool were measured at the protein level. Except for IL-10, all investigated cytokines are associated with a proinflammatory effect. The results are shown in Fig 6 (S8 Fig shows the absolute concentration values). At 24 h after transfection of the M2-like macrophages with the siRNA pool, the cytokine profile had already shifted to an M1-like phenotype in the transfected group compared to the control group (mock treated M2-like macrophages) (Fig 6A). These iM1 macrophages secreted the proinflammatory (M1-like) cytokines IFN-β, IL-1α, IL-6, IL-27, MCP1 and TNF-α but not IFN-γ. In accordance with the M1-like phenotype, the iM1 macrophages did not induce the secretion of IL-23, IL-1β, IL-17A, IL-12p70, IL-10 or GMCSF. Hence, aside from the shift from IFN-γ to IFN-β production in the iM1 macrophages, the iM1 and M1-like macrophages showed the same cytokine secretion pattern. This similarity became even more prominent after prolonged transfection periods, resulting in considerably higher amounts of all aforementioned cytokines upon transcription factor expression knockdown (48 h and 72 h post transfection, Fig 6B and 6C).

To obtain a broader overview of the impact of the regulators with knocked-down expression on the transcriptome of iM1 macrophages, RNA sequencing was performed to obtain the iM1 transcription profile. In addition, we determined the transcription factors maximally contributing to this shift and performed RNA sequencing of M2-like macrophages after knocking down the expression of single genes.

**Fig 7:**
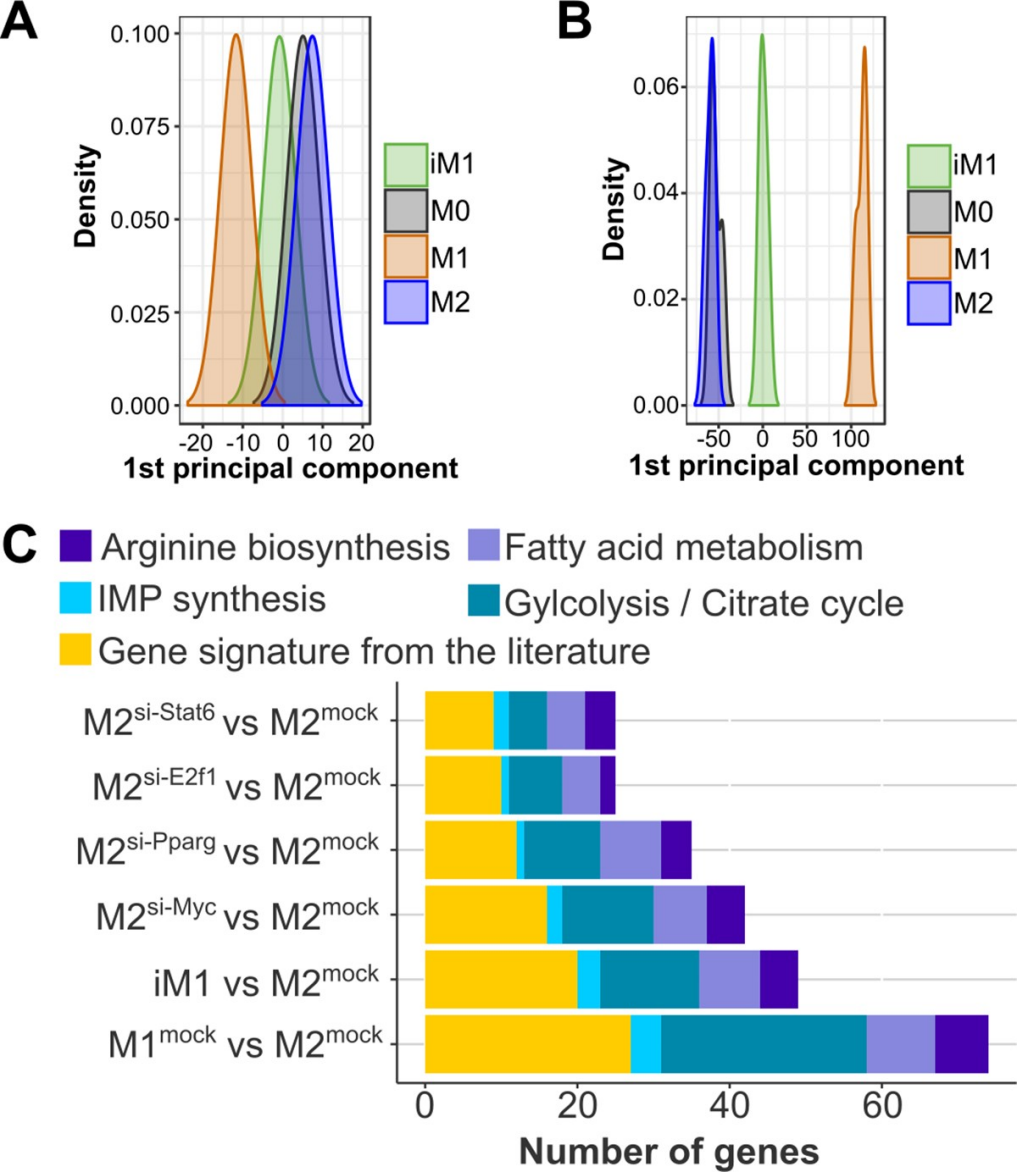
The impact of the predicted regulators on metabolic and M1/M2 associated genes was investigated on a large scale by profiling the transcriptome of iM1 macrophages (reprogrammed M2-like macrophages). The first principal component of our gene signature (n = 112 genes) (A) and all profiled genes (B) show the shift of iM1 from M2-like macrophages towards M1-like macrophages. (C) The reprogramming effect of the predicted regulators on the extended gene signature is visualized as the number of genes that were differentially expressed in the same direction after TF expression knockdown in M2-like macrophages as the expression observed in M1-like macrophages compared to that observed in (mock treated) M2-like macrophages.

The reprogramming of M2-like-polarized macrophages into iM1 macrophages resulted in iM1 macrophages with an expression profile very similar to that of M1-like macrophages (P = 3.04E-09, Fisher’s exact test). Fig 7A shows the first principal component for the gene signature (112 genes) and Fig 7B shows the first principal component for all profiled genes. As shown in Fig 7C and S6 Table, the majority of the genes in the literature signature were affected by our approach of the combined expression knockdown: in the iM1 macrophages, 74% (20 out of 27) of the genes in this signature were correctly reprogrammed, and 62% (29 out of 47) of the metabolic genes were successfully reprogrammed (complete extended gene signature: 49 out of 74, i.e., 66% correctly reprogrammed). Additionally, knocking down the expression of single transcription factors from our list induced a significant switch from an M2-like phenotype towards an M1-like phenotype (Myc: P = 5.68E-07, Pparγ: P = 2.20E-06, E2f1: P = 1.43E-05, and Stat6: P = 8.09E-03, Fisher’s exact test). Knocking down the expression of the predicted transcription factors affected genes involved in central metabolism including arginine synthesis, IMP synthesis, fatty acid synthesis, glycolysis and the citrate cycle. Our results indicated that the reprogramming of metabolic genes was mainly mediated by knocking down the expression of Myc (26 genes when individually knocked down) and Pparγ (23 genes when individually knocked down). We observed decreased expression of tissue repair genes (Chil3 and Retnla) and Tgfb2 (S1 Fig). Consistent with previous findings, the expression of M1-associated cytokines (Il6 and Tnfα) was upregulated in the iM1 macrophages. Regarding the differential expression of all genes (not only those in the focused gene signature), we observed 3,999 and 4,447 genes to be differentially regulated in the M1-like macrophages compared to the M2-like macrophages, respectively, and of these genes, 44% were correctly reprogrammed in the iM1 macrophages (overlap of 1,431 genes with upregulated expression and 2,438 genes with downregulated expression).

In summary, quantitative gene expression and cytokine secretion analysis demonstrated efficient but incomplete *in vitro* repolarization of M2-like macrophages into an iM1 phenotype mediated through knocking down the expression of the predicted regulators E2f1, Myc, Pparγ and Stat6. Combined expression knock down of these regulators reprogrammed more genes in the M1-like direction than individual expression knock down, indicating a cumulative effect of these transcription factors on the regulation of M2-like to M1-like polarization.

## Discussion

We identified differences in metabolic regulation when comparing M1-like- and M2-like-polarized macrophages derived from peritoneal exudate cells (PECs). In the M1-like macrophages, genes involved in immune responses and metabolic processes such as nitric oxide synthesis and glycolysis, respectively, exhibited upregulated expression. Aerobic glycolysis, also known as the “Warburg effect”, has been described as an exploitative process in which ATP is produced rather lineally by converting glucose into lactate [23]. In addition to occurring in tumor cells, the Warburg effect has also been observed in immune cells [24–27]. We noted upregulated expression of all genes involved in the aerobic glycolysis pathway from hexokinases to lactate dehydrogenase in the M1-like macrophages. This enhanced gene expression pattern enables fast energy supply, which is needed for cytokine production and effective eradication of invading pathogens. This is in line with observations in several studies that shifting to glycolysis and away from the TCA cycle lead to more inflammatory macrophages [28,29]. ONeill and Pearce suggested that aerobic glycolysis enables induced NADPH production, either via the pentose phosphate pathway or via malic enzyme and pyruvate, to produce NO via iNOS or to produce other radical oxygen species [30]. Similarly, Jha *et al*. observed increased glycolytic activity and reduced oxidative phosphorylation activity in bone marrow-derived macrophages upon stimulation with LPS and IFNγ [10]. In turn, the M2-like macrophages showed rather upregulation of oxidative phosphorylation and anabolic metabolic processes such as amino acid and nucleotide biosynthesis, and, fatty acid metabolism was upregulated. ONeill and Peace suggested that glycolysis suits M1 for fast, short-termed activation affecting bacteria or fungi, whereas oxidative processes may rather support the survival of M2 macrophages during their longer and persisting combat with parasitic helminth infections [30].

Next, we sought to determine the regulators responsible for the metabolic and functional reprogramming of the M2-like macrophages. To focus our reprogramming efforts, we assembled a gene signature comprising 112 genes from the central metabolic pathways with the most differences between the M1- and M2-like macrophages as well as M1- and M2-associated genes from the literature. Employing the transcription profiles of the M1- and M2-like macrophages and generic chromatin binding information from databases, we generated a mixed integer linear programming-based gene regulatory model followed by a statistical analysis pipeline and determined a refined list of four transcription factors (E2F1, MYC, PPARγ and STAT6) predicted to be responsible for the differential gene regulation between the M1- and M2-like macrophages. By knocking down the expression of all four regulators in M2-polarized macrophages, we induced upregulated expression of M1-associated genes and downregulated expression of M2-associated genes. We observed that 66% of the genes were correctly reprogrammed i.e., differentially expressed in the same direction as that observed in M1-like macrophages (mock treated) compared to that observed in M2-like macrophages (mock treated). In addition, 13 secreted cytokines were investigated. Both, the gene expression levels and the secreted amounts of all proinflammatory cytokines analyzed, except IFNγ, were increased in the reprogrammed macrophages, again indicating the switch to an M1-like phenotype. Instead of higher secretion of IFNγ, higher secretion of IFNβ was observed in the reprogrammed macrophages compared with the control M2-like macrophages. This secretion pattern contrasted with the observed secretion pattern in the M1-like macrophages but is actually in agreement with a recent report in which M1-like macrophages showed increased IFNβ secretion [31]. Overall, we observed that 8,446 genes were differentially expressed between M1-like and M2-like macrophages. In our reprogrammed iM1 macrophages, 44% of these genes showed correct differential expression. It thus would be intriguing to expand these studies in future projects. Hence, the experimental validations confirmed that our predicted regulators were essential for maintaining the M2-like phenotype, and moreover, the inhibition of these regulators allowed M2-like macrophages to be driven into an activation state similar to that of M1-like macrophages.

All predicted transcription factors were active in M2-like macrophages, and siRNA-mediated knockdown repressed their transcription and hence their activity. Interestingly, the expression of cell cycle regulators such as MYC and E2F1 needed to be knocked down to achieve reprogramming, suggesting that enforced cell cycle arrest in M2-like macrophages might stimulate the acquisition of an M1-like phenotype. Indeed, from the other direction, it has been shown that intercalator-induced DNA damage reprograms white blood cells into tolerance or resilience in a murine sepsis model [32]. In activated macrophages, multiple and distinct roles of the cell cycle have been observed in M1- and M2-like macrophages. Considering M2-like macrophages, the simultaneous injection of IL-4 and thioglycollate into the murine peritoneal cavity triggers rapid proliferation in tissue-resident macrophages *in situ* [33]. In turn, it has been observed that in M1-like macrophages, the gene expression of immunostimulatory MHC class II molecules is regulated by the cell cycle [34].

Furthermore, we estimated the influence of each of the predicted regulators by targeting E2f1, Myc, Pparγ and Stat6 individually. Increased gene expression of inflammatory cytokines after individually knocking down Myc expression showed that this transcription factor is a potential inhibitor of M1-like activation. Hao and coworkers showed that transfecting M2-like macrophages with shRNA-cMyc leads to reduced expression of Arg1 and Mrc1, indicating that MYC may facilitate the maintenance of the M2-polarized state [35]. This study is in line with a study by Pello and coworkers that reported that M2-like polarization is dependent on c-Myc expression [36]. After knocking down Myc expression, we observed reprogrammed metabolic pathways such as glycolysis and IMP biosynthesis. MYC has been described as a master regulator of cell metabolism, including nucleotide biosynthesis [37]. Particularly, nucleotide metabolic genes are enriched among MYC targets [38], and IMP dehydrogenases have been described as direct targets of MYC [39], supporting our observation of downregulated IMP synthesis after Myc expression knockdown. Limited information is available about the specific function of E2F1 in M2-polarized macrophages. We observed that knocking down E2f1 expression on its own induced an M1-like expression pattern in our extended gene signature, and particularly, the observed M1-like and M2-like associated genes from the literature signature suggested an anti-inflammatory role for E2f1 in M2-like macrophages. After E2f1 expression knockdown, the levels of glycolysis genes encoding hexokinases 1–3 and inflammatory genes (i.e., Ccl2, Ccl3, and Cxcl9) were upregulated, while fatty acid metabolism-related gene expression was downregulated. Further evidence of an anti-inflammatory function of E2f1 is provided by a study reporting enhanced levels of IL-12p70 and TNF-α in dendritic cells after knocking down the expression of E2f1 [40]. However, different effects of E2F1 on macrophage function were described previously by another study. The RNAi-mediated inhibition of E2F1 in J77A4.1 macrophage cells stimulated with LPS resulted in reduced IL-6 and TNF-α secretion. Likewise, diminished levels of TNF-α, IL-1β and IL-6 were observed in bone marrow-derived macrophages from E2F1-deficient mice three hours after systemic LPS challenge. Furthermore, decreased production of TNF-α and IL-6 was found after stimulation with six different TLR ligands [41]. Thus, the inflammatory and anti-inflammatory roles of E2F1 appear to be complex and need further investigation.

We observed that Pparγ and Stat6 regulate immune-related pathways such as cell adhesion and antigen processing and presentation. Previous research showed that IL-4-induced Stat6 expression mediates the inhibition of Nos2 expression in a murine macrophage cell line [42]. Likewise, we observed that Nos2 expression was downregulated after individual and combined expression knockdown of E2f1, Myc, Pparγ and Stat6. Notably, Stat6 also negatively regulates IFN-γ-induced Stat1 expression directly and indirectly [43–45]. Furthermore, our results showed that all four identified transcription factors, i.e., E2F1, MYC, PPARγ and STAT6, had an impact on the regulation of fatty acid metabolism, i.e., the downregulation of the expression of one transcription factor led to a decrease in this metabolic process. This result is in line with the recent observation that M2-like macrophages of STAT6 null mice failed to upregulate the expression of metabolic genes involved in fatty acid oxidation [46]. In another study, decreased arginase activity and increased IL-6 production were also reported in macrophage-specific Pparγ knockout mice [47].

In summary, we postulate that targeting E2f1, Myc, Pparγ and Stat6 in M2-like macrophages induces a shift towards immunostimulatory M1-like macrophages, which may support immune defense and hence improve therapy against infection. In several studies, it has been observed that induced shifts between tolerance and resistance can be beneficial. It is known that tumors in septic patients can regress. E.g., in a patient with stage IV cutaneous metastatic melanoma, complete tumor regression was observed after multifactorial sepsis during chemotherapy. After targeted antibiosis and combined complication-free chemotherapy, the clinical condition of the patient improved, resulting in the complete clearance of metastases by end of therapy [48]. Furthermore, several studies have reported beneficial effects from anti-inflammatory therapies; macrolides, in addition to their antibiotic effect, can modulate the immune response and are applied to treat chronic inflammatory diseases such as panbronchiolitis, bronchiectasis, rhinosinusitis and cystic fibrosis. The immunomodulatory mechanisms underlying the effects of macrolides, although not completely understood, may be due to the inhibition of MAPK/ERK signaling and NF-κB. Macrolides accumulate within cells, suggesting that they may associate with the receptors or carriers responsible for the regulation of the cell cycle and immunity (see review by Kanoh and Rubin (2010) [49]). In line, it is known that activation of NF-κB can lead to M1-like polarization (see review [50]). Indeed, we observed a significant higher activity (derived from the expression of the target genes, see Materials and Methods) of NF-κB in iM1 and M1-like compared to M2-like macrophages (P = 0.0072 and P = 0.025, respectively, Student’s T-test). Investigating the complementary and synergistic effects of macrolides with E2f1, Myc, Pparγ and Stat6 may provide a better understanding of these beneficial mechanisms and may lead to developments modulating their mode of action. For the future, we suggest to substantiate the shift in polarization also metabolically, e.g. in the consumption and production of metabolic compounds and oxygen consumption. The problem of trying to reprogram M2-like into M1-like macrophages is mathematically symmetric to the shift from M1-like to M2-like macrophages. The latter would have clinically highly relevant applications, e.g. reducing the inflammatory response during the acute phase of sepsis. Experimentally, such an analysis was out of scope of this study, but can be fruitful in future work. Methodologically, we inferred metabolic fluxes from gene expression employing a mixed integer programming based model linearly correlating the metabolic fluxes with the expression of the according enzyme coding gene(s). Several other methods have been published with a similar aim, i.e. to predict metabolic fluxes from gene expression [51–53]. A CPU demanding, but necessary task for these approaches is to reduce thermodynamically infeasible loops. As a future aspect it would be intriguing to compare these methods which may lead to an improved model better reflecting the metabolic fluxes.

## Conclusions

Understanding and manipulating the polarization of macrophages to shift these cells from an immunosuppressive state into an immunostimulatory state or *vice versa* have a high potential to treat a broad range of widespread diseases such as sepsis, cancer or chronic inflammatory diseases. The present study provides new molecular insights into the reprogramming of macrophages. Using siRNA pools targeting E2f1, Myc, Pparγ and Stat6, we demonstrated that these transcription factors are essential to maintaining an M2-like phenotype. The reprogramming of M2-like macrophages by repressing these transcription factors induced a switch towards an M1-like phenotype as evidenced by i) increased secretion of proinflammatory cytokines, ii) a characteristic gene expression signature and an extended gene signature based on previously described M1/M2 marker genes and central metabolism genes, and iii) an overall differential expression pattern similar to that of M1-like macrophages. Our results may support the design of immunotherapy-based strategies targeting macrophages to modulate the immune system in widespread chronic and acute diseases.

## Materials and methods

### Isolation of murine peritoneal exudate cells (PECs)

C57BL/6 mice (H2^b^, aged 8–20 weeks) were obtained from Charles River (Sulzfeld, Germany) and housed under SPF conditions in individually ventilated cages (IVCs) at the animal facility of the DKFZ. C57BL/6 mice were injected intraperitoneally (i.p.) with 1 ml of 3% (w/v) thioglycolate containing medium (Applichem, Darmstadt, Germany; Cat. No. A3869) using a 27 G needle. Four days later, mice were sacrificed by gradual CO2 exposition and PECs were isolated upon lavage of the peritoneum with 8 ml ice cold PBS. This procedure yielded approximately 1.5 x 10^7^ PECs per mouse.

### Ethics statement

Animal experiments were approved by the Internal Ethics Committee of the DKFZ and by the District Government in Karlsruhe, Germany (approval ID 35–9158.81/G-211/16).

### *In vitro* polarization of PECs

PECs were maintained in Dulbecco's modified Eagle's medium (DMEM) containing 10% fetal calf serum (FCS), and 2 x 10^6^ cells were seeded into each well of a 6-well plate. After washing with phosphate-buffered saline (PBS), adherent cells were used for this study. Next, the cells were cultured for 24h in DMEM supplemented with either 10 ng/ml IL-4 or 100 ng/ml LPS and 50 ng/ml IFN-γ to induce M2-like or M1-like polarization, respectively. Within the whole article, we are referring to these macrophages derived from PECs when we write "macrophages". Then, PECs were transfected with siRNA pools targeting Myc (Dharmacon, Lafayette, USA), Ctcf, E2f1, Pparγ, and Stat6 (siTools Biotech, Planegg, Germany) by using DharmaFECT 4 Transfection Reagent according to the manufacturer’s instructions. No effects on viability were observed following siRNA transfection as judged by microscopic inspection. In the reprogramming experiments, PECs were polarized for 24 h into M2-like macrophages using IL-4 and subsequently transfected with siRNA pools targeting Myc (Dharmacon, Lafayette, USA), Ctcf, E2f1, Pparγ, and Stat6 (siTools Biotech, Planegg, Germany) by using DharmaFECT 4 Transfection Reagent according to the manufacturer’s instructions (subsequent culturing took place in the absence of IL-4). Cytokine assays, quantitative real-time PCR and RNA sequencing were performed 24, 48 or 72 h post transfection. RNA sequencing experiments were performed 24h after polarization of PECs (obtaining the transcription profiles of M0, M1-like and M2-like macrophages, without reprogramming or transfection) and 48h post transfection (for the reprogramming study). PECs treated only with the transfection reagent were used as mock controls.

### Quantification of secreted cytokines and chemokines

Secreted cytokine levels in PEC culture medium were measured by using the LEGENDplex Mouse Inflammation Panel (13-plex, with Filter Plate, Biolegend, Cat No. 740150; Lot No. B242042) according to the manufacturer’s protocol with double the sample volume and sample incubation time at 4°C overnight.

### RNA isolation and quantitative real-time PCR

RNA extraction from PECs (RNA extraction from PECs (n = 3 biological replicates for M0, M1-like, M2-like and iM1 macrophages) was carried out using the RNeasy Plus Mini Kit according to the manufacturer’s instructions (including on-column DNA digestion). The extracted RNA was purified by ethanol precipitation prior to reverse transcription using the Transcriptor First Strand cDNA Synthesis Kit according to the manufacturer’s instructions. Gene expression was measured using quantitative real-time PCR. Therefore, 2 X Power SYBR Green PCR Master Mix (Thermo Fisher Scientific, Waltham, USA; Cat. No. 4367659), 10 ng of cDNA, 400 nM primer pair and nuclease-free water were mixed to a total volume of 20 μl. The selected genes were amplified using the ABI 7300 Real-time PCR System. All the obtained qPCR data were normalized to the data for the housekeeping gene Rpl19. Primers were purchased from Sigma-Aldrich (St. Louis, USA), dissolved in ddH_2_O to a stock concentration of 100 μM and stored at -20°C. A list of all primers used is given in S7 Table.

### RNA sequencing and analysis

RNA was extracted using the Qiagen RNeasy Plus Mini Kit according to the manufacturer’s instructions. Samples were sequenced by the Genome and Proteomics Core Facility of the German Cancer Research Center in Heidelberg (HiSeq SE50) and Novogene (Hong Kong, HiSeq PE150). The resulting reads were trimmed and mapped against the mouse GRCm38/mm10 reference genome using Trimmomatic [54] and TopHat [55], respectively. Read counts were calculated using featureCounts [56,57]. DESeq2 [58] was used to identify the genes with differential expression between M1-like- and M2-like-polarized macrophages. To note, throughout the article, we refer to "up- and down-regulated genes" in respect to the comparison in gene expression between M1-like (or later induced M1, iM1) and M2-like macrophages, irrespective to the expression in M0 macrophages.

Gene set enrichment analysis was performed using g:Profiler [59] and the gene set definitions of Gene Ontology (GO) and KEGG analyses. The enriched gene sets from the GO analysis were filtered with customized scripts to reduce redundant GO terms using linear optimization. In detail, redundancy between two gene sets was quantified using Jaccard similarity coefficients,

$$J(A,B)=\frac{\left|A\cap B\right|}{\left|A\cup B\right|}$$ (1.1)

in which *A* and *B* are gene sets containing significantly differentially expressed genes. An undirected graph *G* = (*X*, *E*) is introduced, with *X being gene sets* as vertices and *E being gene set pairs with J(A*,*B) > = 0*.*5* as edges of the graph. A mixed integer linear model (weighted stable set problem) was set up with a constraint for each edge to select at most one of the vertices of an edge:

$$Max\;\sum_{i=1}^{n}w_{i}X_{i}$$ (1.2)

subjected to

$$X_{i}+X_{j}\leq 1\;\mathrm{for}\;\mathrm{every}\;\left\{i,j\right\}\;\epsilon \;E\;\mathrm{and}\;X_{i}\;\epsilon \;\{0,1\}\;\mathrm{for}\;1\leq i\leq n,$$ (1.3)

where, w_i_ is the weight of a gene set. The weight is derived from its significance value (p-value) and calculated as 1 − log_10_(p-value)/100. This maximization was done employing linear integer programming solved by the software Gurobi (version 7.5.2 https://www.gurobi.com). This led to an optimal selection of at most one gene set from a pair in such a way that the overall number of non-redundant gene sets were maximized. The remaining gene sets from the GO analysis, together with the gene set definitions from the KEGG analysis, were grouped by manual curation into broader categories comprising anabolic processes (ANP); nucleic acid metabolism (NAM); oxidative stress (OXS); metabolic processes (MEP); protein transport, modification and processing (PRO); transport (TRA); catabolic processes (CAP); the damage response (DAR); cellular death (CED); cellular growth (CEG); development (DEV); response to stimuli (RES); signaling processes (SIP); the immune response (IMR); disease-related pathways (DIS) and miscellaneous (MIS). Raw data and read counts have been deposited in the Gene Expression Omnibus repository (GSE 129253).

### Flux balance analysis

Our metabolic network was based on a macrophage cell line model (RAW 264.7 cells) from Bordbar *et al*. [20]. A constraint for the minimal biomass production (biomass ≥ 0.028) was set as reported by Bordbar *et al*. To note, the lower bound on the biomass reaction was set by Bordbar and coworkers to the lower experimental growth rate of 0.0281/h to mimic minimal maintenance of the macrophages. The model of Bordbar et al. bases on proteomics data from another study in which macrophages were grown in Dulbecco’s modified Eagle’s medium (DMEM) supplemented with 10% heat-inactivated fetal bovine [60]. Further, the reaction

$$Ornithine+Carbamoyl‐phosphate⇌H^{+}+P_{i}+L‐Citrulline$$ (2)

catalyzed by ornithine carbamoyltransferase was added to the network. We predicted the flux of a set of reactions for different macrophage phenotypes. In our model, a gene expression-based flux ${\tilde{v}}_{r,p}$ of reaction r and condition p was derived from a linear model in which the expression of the gene encoding the corresponding enzyme, ${\bar{g}}_{r,p}$, was related to flux ${\tilde{v}}_{r,p}$ by

$$|{\tilde{v}}_{r,p}|={minV}_{r}+({\bar{g}}_{r,p}-{minG}_{r})∙\frac{{maxV}_{r}-{minV}_{r}}{{maxG}_{r}-{minG}_{r}}$$ (3.1)

where ${\bar{g}}_{r,p}$ was the Z-transformed gene expression value of macrophage type *p ϵ* {*M*0,*M*1,*M*2} and *minG*_*r*_ and *maxG*_*r*_ were the lowest and highest Z-transformed gene expression values for reaction *r* across the conditions. *maxV*_*r*_ and *minV*_*r*_ were the lowest and highest absolute fluxes and were determined by flux variance analysis [61]. Metabolic fluxes were constrained by the known stoichiometry of the reactions *r* and their substrates and products, compiled into the stoichiometric matrix *S*, and constrained by lower (*lb*_*r*_) and upper (*ub*_*r*_) bounds for each reaction *r*. Flux variance analysis [61] was performed to determine the lower and upper bounds for all reactions. Assuming that the metabolic network is in a steady state, in which the metabolite concentrations do not change over time, yields the constraints (3.2)

$$S_{r}∙v_{r,p}=0,$$ (3.2)

and flux ${\tilde{v}}_{r,p}$ is constrained by

$${lb}_{r}\leq v_{r,p}\leq {ub}_{r}.$$ (3.3)

*S*_*r*_ is the stoichiometric matrix, and *v*_*r,p*_ is the flux of reaction *r* and macrophage phenotype *p*. The objective function for the optimization problem was to minimize the difference between the gene expression-based flux prediction ${\tilde{v}}_{r,p}$ and the predicted flux *v*_*r*,*p*_:

$$\mathrm{Minimize}\;\sum_{r,p}w_{r,p}∙|\left|v_{r,p}|-|{\tilde{v}}_{r,p}\right||,\;\mathrm{with}$$ (4.1)

$$w_{r,p}=\left\{ \begin{array}{c} \frac{100}{{maxV}_{r}∙n}\;\forall r\in CR\\ \frac{1}{{maxV}_{r}∙n}\;otherwise \end{array}\right.$$ (4.2)

where *maxV*_*r*_ is the highest absolute flux of reaction *r*, *n* is the number of genes associated with reaction *r*. Hence, our model did not consider AND/OR rules if more than one gene was associated with a reaction *r*. We rather calculated the associated flux by averaging the gene expression values using the weights *w*_*r*,*p*_ for each gene encoding this reaction. Reactions that were not associated with any genes were not considered in the objective function. *CR* denotes the set of core reactions associated with the pathways of interest (glycolysis, citrate cycle, urea cycle, or IMP synthesis). The fluxes of the core reactions (*r* ∈ *CR*) were of special interest and thus were weighted by 100. Lower weights (= 1) were used for reactions that were direct neighbors of the core reactions. All other reactions were not part of the objective function. The flux of each reaction *r* was normalized by the maximum absolute flux value derived from flux variance analysis yielding the weights *w*_*r*,*p*_ (formula 4.2). In addition, we developed a method to detect and remove the thermodynamically infeasible loops that were part of the flux solution space. First, the flux vector *v* of the optimal solution for the system (1) to (4.2) is required. *Supp*(*v*) is the support of *v* and contains a subset of the reactions, where *v* is higher than or equal to the threshold 0.01. 0.01 was used as the best trade-off between CPU time and getting reasonable models. Next, the length of a minimum-containing cycle of the support is determined by solving the following MIP problem:

$$\mathrm{Minimize}\;\sum_{r}\lambda_{r}$$ (5.1)

Subject to

$$\sum_{r}S_{r}∙\lambda_{r}=0$$ (5.2)

$$\lambda_{r}\geq {inFC}_{r}$$ (5.3)

$$\sum_{r}{inFC}_{r}\geq 2$$ (5.4)

in which *S*_*r*_ is the stoichiometric matrix and *λ*_*r*_ indicates the flux of reaction *r* (∀*r* ∈ *Supp*(*v*)). *inFC* is a binary variable that equals 1 if and only if the reaction *r* is involved in the detected thermodynamic cycle. Notably, all sums in (5.1) to (5.4) are built over *Supp*(*v*). If there is no thermodynamically infeasible loop, then this MIP problem is infeasible, and we can stop. Otherwise, there are at least two reactions involved in a thermodynamic cycle. The corresponding variables ${inFC}_{r_{1}}$, …, ${inFC}_{r_{k}}(k\geq 2)$ are all equal to 1. In this case, we forbid this cycle by the constraint

$$\sum_{i=1}^{k}{inFC}_{r_{i}}\leq k-1.$$ (6)

This inequality is added to the systems (1) to (4.2), which enforces that after the next optimization, at least one flux of the corresponding cycle will be 0, but which is 0 is decided by the optimization. This procedure of adding inequalities of type (6) was repeated until all thermodynamically infeasible loops were detected and forbidden. So first we prefer small loops to be detected. This is to decrease the overall running time. Afterwards the detected loop is forbidden by inequality (6) and we look for the next loop by reoptimization. After the optimization leads to no feasible solution any more, there is no loop in the solution (with support ≥ 0.01). Otherwise a solution would have been found.

### Modeling of regulatory networks and *in silico* reprogramming

We selected 112 metabolic genes and M1/M2-associated genes from the literature for which we predicted a short list of transcription factors best explaining the transcription profiles of these genes. We collected evidence-based interactions for all transcription factors from various sources: MetaCore (Thomson Reuters), ChEA [62], ECRbase [63], Mouse ENCODE [64,65], and TfactS [66,67]. We compiled transcription factor binding information from MetaCore, ChEA [62] and ENCODE [64,65]. Additionally, we added the two additional databases ECRBase and TfactS. ECRBase is based on alignments of evolutionarily conserved transcription factor binding sites [63]. TfactS contains interaction information inferred from the regulation of transcription factors in the gene expression data of experimentally well-characterized target genes listed in TRED [68], TRRD [69], PAZAR [70] and NFIregulomeDB [66,71]. Interaction information from MetaCore labeled "direct" was regarded as the most reliable and hence weighted by a factor of two. If an interaction was listed in two out of the (a) MetaCore (labeled "unspecified"), (b) ChEA and (c) ECRbase databases, it was weighted by one (for each source). A listed entry in mouse ENCODE was weighted by 0.5. The interactions in TfactS were considered to be of weaker evidence and were weighted by 0.25. This approach led to the overall edge score ES_t,i_ of transcription factor *t* and target gene *i*. If ES_t,i_ was larger than zero, the interaction was considered to be known. We considered only transcription factors for which we found known interactions with at least 15 genes in our gene set. In addition, the activity for each transcription factor was determined as described previously [22]: the activity of a regulator was calculated from the average expression (z-scores) of its known target genes. We predicted the regulators for a set of genes with a linear approach by minimizing the difference between the predicted gene expression values ${\tilde{g}}_{i,k}$ and the measured gene expression values *g*_*i*,*k*_ of gene *i* in sample *k* [72]:

$$\mathrm{Minimize}\;\sum_{i,k}\left|\left|g_{i,k}|-|{\tilde{g}}_{i,k}\right|\right|,\;\mathrm{with}$$ (6.1)

$${\tilde{g}}_{i,k}=\beta_{0,i}+\sum_{t}\beta_{t,i}∙{ES}_{t,i}∙{act}_{t,k}$$ (6.2)

where *t* is the transcription factor potentially regulating gene *g*_*i*_ and *act*_*t*,*k*_, is the above-described activity of transcription factor *t* in sample *k*. ES_t,i_ is the edge score of transcription factor *t* to gene g_i_. The beta values were the parameters to optimize. *β*_*t*,*i*_ is the parameter to optimize the impact of *t* regulating gene *i*, *β*_0,*i*_ is an additive offset for gene *i*. We applied this method within the following bootstrapping scheme. First, we randomly selected 10 genes out of the gene set and predicted their regulators within a 3-fold cross-validation using the described regression model (Eqs 6.1 and 6.2). To avoid overfitting, the maximum number of regulators in the model was set to 5. After 1,000 repetitions, we collected the predicted transcription factors from all runs and performed Fisher’s exact tests to determine the transcription factors that were selected significantly more often than by chance. These transcription factors were used in the last step in which we predicted the most parsimonious set of reprogramming transcription factors for the whole gene set within a 3-fold cross-validation using the same regression scheme (Eqs 6.1 and 6.2) with at most five regulators. For reprogramming the macrophages from the M2-like to M1-like phenotype (*M*2→*M*1) *in silico*, we replaced the activity (*act*_*t*,*M*1_) of the selected transcription factors in the model of the gene of interest as follows:

$${\tilde{g}}_{i,M2\to M1}=\beta_{0,i}+\sum_{t}\beta_{t,i}∙{ES}_{t,i}∙{act}_{t,M1}+\sum_{u}\beta_{u,i}∙{ES}_{u,i}∙{act}_{u,M2}$$ (7)

where *i* ∈ the genes of the extended gene signature, *t* ∈ the selected transcription factors (Myc, E2F1, Pparg, and Stat6), and u ∈ all other transcription factors in the model, i.e., leaving the rest of the model as an M2-like model. All betas were used from the predicted M2-like models.

## Supporting information

S1 Fig Gene expression profiles of the extended gene signature in (mock treated) M1 macrophages or M2 macrophages treated with siRNA-pools targeting E2f1, Myc, Pparg, Stat6 and their combination, compared to (mock treated) M2 macrophages. Significantly upregulated genes with a log2 fold change ≥ 1.5, non-significantly upregulated genes, significantly downregulated genes with a log2 fold change ≤ -1.5, and non-significantly downregulated gens are shown in red, orange, blue and light blue, respectively.
(TIF)

S2 Fig Gene regulatory network including the short list of four transcription factors (E2f1, Myc, Pparg, Stat6) and 112 genes comprising 76 metabolic genes from biochemical pathways being differentially regulated in M1 and M2, and a literature derived gene signature comprising of 36 M1 or M2 associated genes. (TIF)

S3 Fig Gene regulatory network including five transcription factors (depicted in black ellipses) and 112 genes comprising 76 metabolic genes from biochemical pathways being distinctively differentially regulated in M1 and M2, and a literature derived gene signature comprising of 36 M1 or M2 associated genes. (TIF)

S4 Fig Performance of the different combinations of the predicted transcription factors was investigated by reprogramming M2 macrophages to M1 macrophages *in silico*. (TIF)

S5 Fig Transcriptional changes of M1 and M2 associated genes after knockdown of five selected TFs were experimentally determined by quantitative PCR. Shown are log2 fold changes in gene expression of 15 genes (from the initial, literature derived signature) in M2 macrophages after treatment with the combined siRNA pool targeting E2f1, Myc, Pparg, Stat6 and Ctcf relative to M2 mock treated macrophages. Samples were extracted 24 h, 48 h and 72 h after siRNA treatment. Error bars are based on the standard error of technical replicates. Gene expression profiles were considered as successfully reprogrammed if a switch towards an M1-like phenotype was observed in both biological replicates (replicate 1 and 2 in grey and black, respectively).
(TIF)

S6 Fig Knockdown efficiency 24 h, 48 h and 72 h after siRNA transduction targeting the transcription factors E2f1, Myc, Pparg and Stat6. Error bars indicate the standard error of three technical replicates.
(TIF)

S7 Fig Transcriptional changes of M1 and M2 associated genes after macrophage polarization to the M1-like phenotype were determined by quantitative PCR. Shown are log2 fold changes in gene expression of 15 genes (from the initial, literature derived signature) in M1-like (24h polarization) and iM1 macrophages (24h post transfection) relative to their expression in M2-like macrophages (24h polarization). Error bars are based on the standard error of technical replicates.
(TIF)

S8 Fig The quantification of cytokines released by M2-polarized macrophages upon transfection with siRNA pools was performed by multiplex analysis. Shown are the concentrations of the cytokines in the medium after 24 hrs (A), 48 hrs (B) and 72 hrs (C), averaged from two technical replicates.
(TIF)

S9 Fig Transcriptional changes in M0 macrophages of M1-like- and M2-like-associated genes after knocking down the expression of four selected TFs were experimentally determined by quantitative PCR. Shown is the differential expression of the genes 72 hrs after siRNA transfection. 72 hrs correspond to a similar culturing time of the cells as if we polarize for 24hrs and then transfect with siRNA for 48hrs. Error bars indicate standard deviations of technical replicates.
(TIF)

S1 Table Results from the differential expression analysis between M1 and M2 macrophages. DESeq2 was used to determine the differentially expressed genes.
(XLSX)

S2 Table Significantly enriched gene sets in M1-polarized *versus* M2-polarized macrophages. Gene set enrichment analysis was performed using g:Profiler followed by filtering of enriched gene sets with customized scripts.
(XLSX)

S3 Table Predicted metabolic fluxes using the constraint based model. (XLSX)

S4 Table Gene signature for M1 and M2 macrophages. Various macrophage subsets had been classified in other studies according to their transcriptional signatures. We assembled a signature from these studies and found very similar differential gene expression (n = 33 agreed, n = 3 disagreed) when comparing expression profiles (M1 *versus* M2 macrophages) of our experiments to the reported gene expression profiles in literature.
(PDF)

S5 Table Transcriptional changes of M1 and M2 marker genes from the literature signature after transfection with the siRNA pool targeting E2f1, Myc, Pparγ and Stat6 (inducing iM1). (PDF)

S6 Table Differential expression of the genes from the extended gene signature. (PDF)

S7 Table Primers for quantitative real-time PCR. Primers were purchased from Sigma-Aldrich (St. Louis, USA), resolved in ddH_2_O to a stock concentration of 100 μM and stored at -20°C. (DOCX)
