# Supplementary figures and images for: Reprogramming of macrophages employing gene regulatory and metabolic network models
Source: PLoS Comput Biol. 2020 Feb 25;16(2):e1007657. doi: 10.1371/journal.pcbi.1007657 (PMC7059956; doi:10.1371/journal.pcbi.1007657)

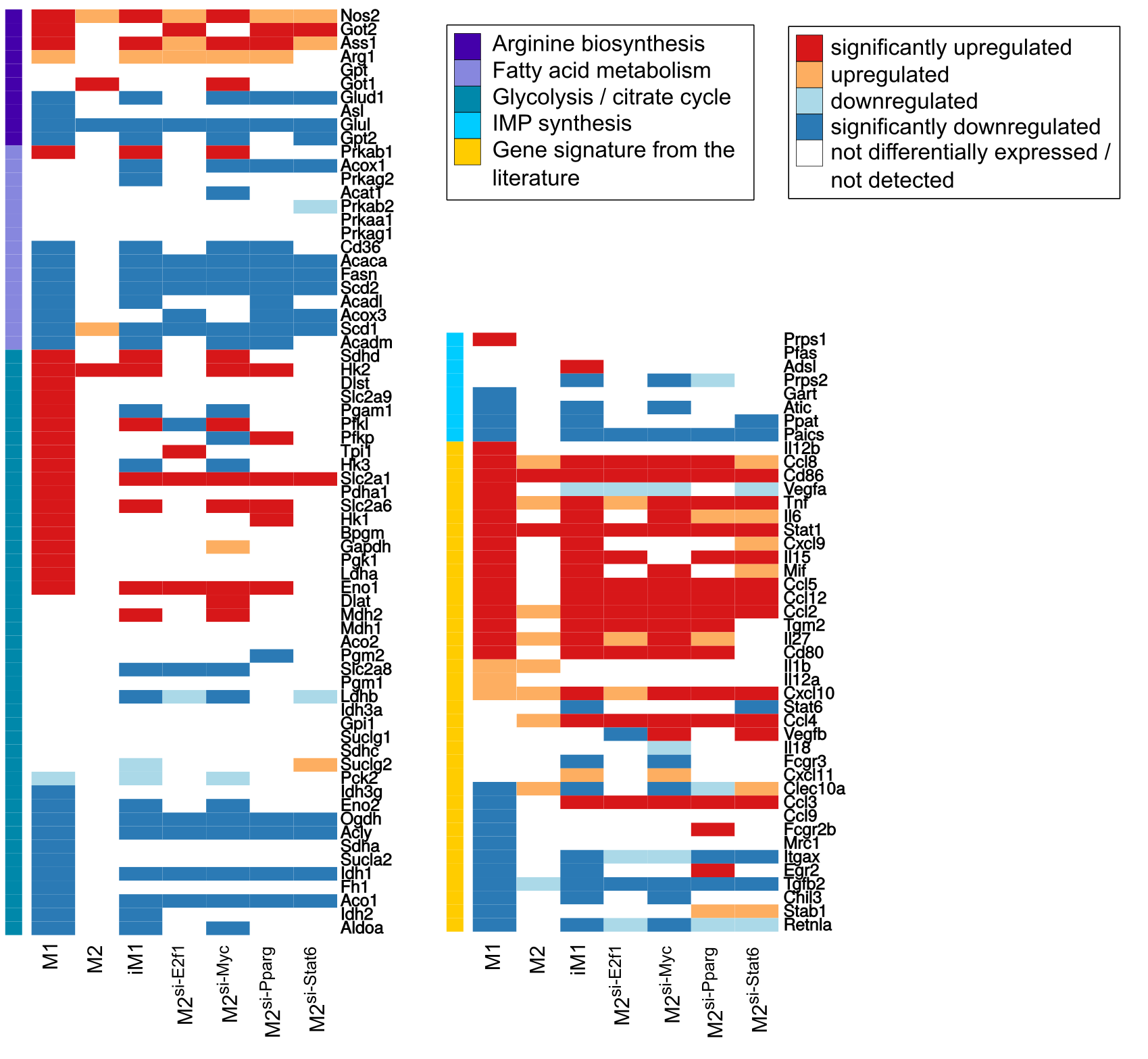

Supplement: S1 Fig — Significantly upregulated genes with a log2 fold change ≥ 1.5, non-significantly upregulated genes, significantly downregulated genes with a log2 fold change ≤ -1.5, and non-significantly downregulated gens are shown in red, orange, blue and light blue, respectively. (TIF) [file pcbi.1007657.s001.tif]

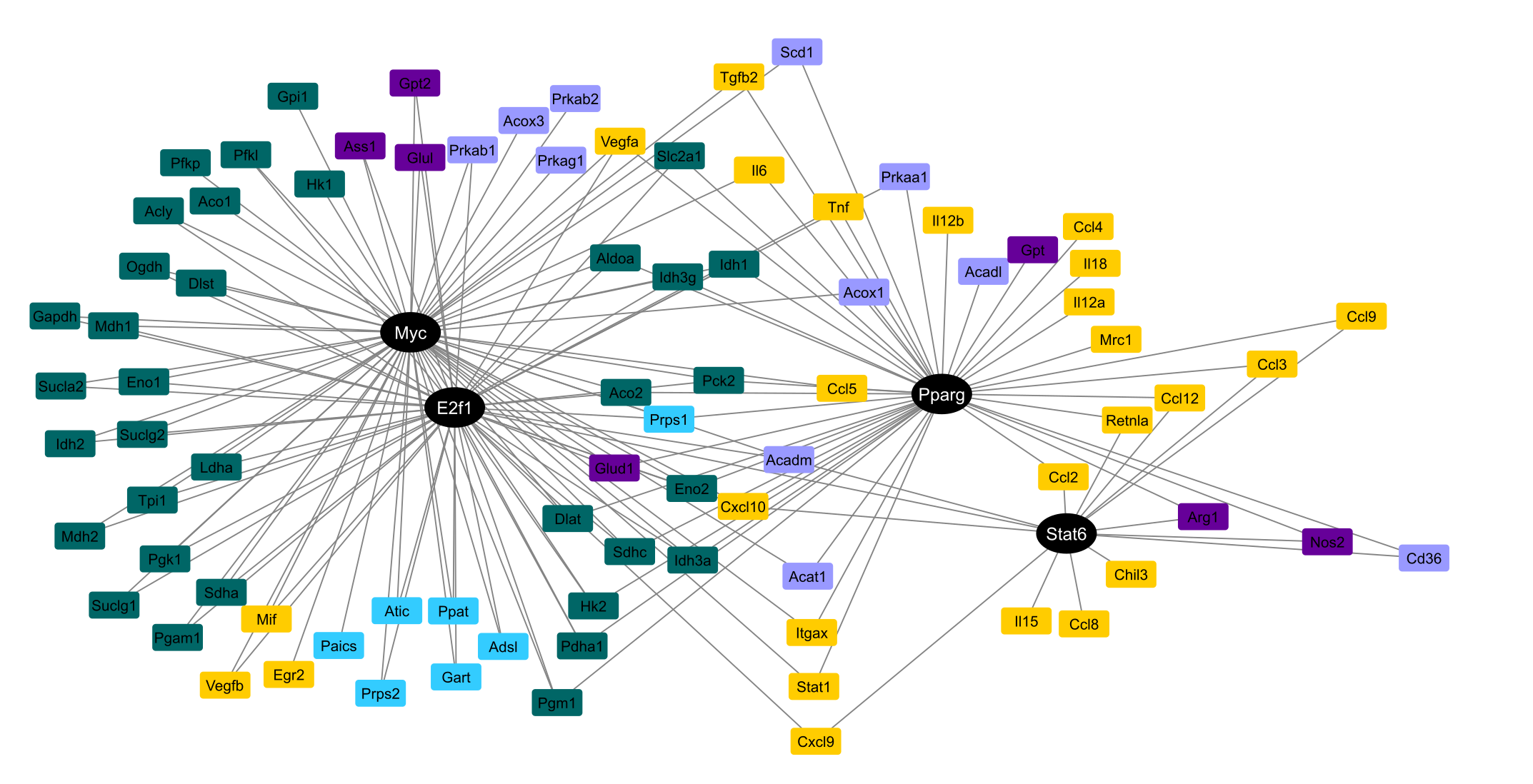

Supplement: S2 Fig — (TIF) [file pcbi.1007657.s002.tif]

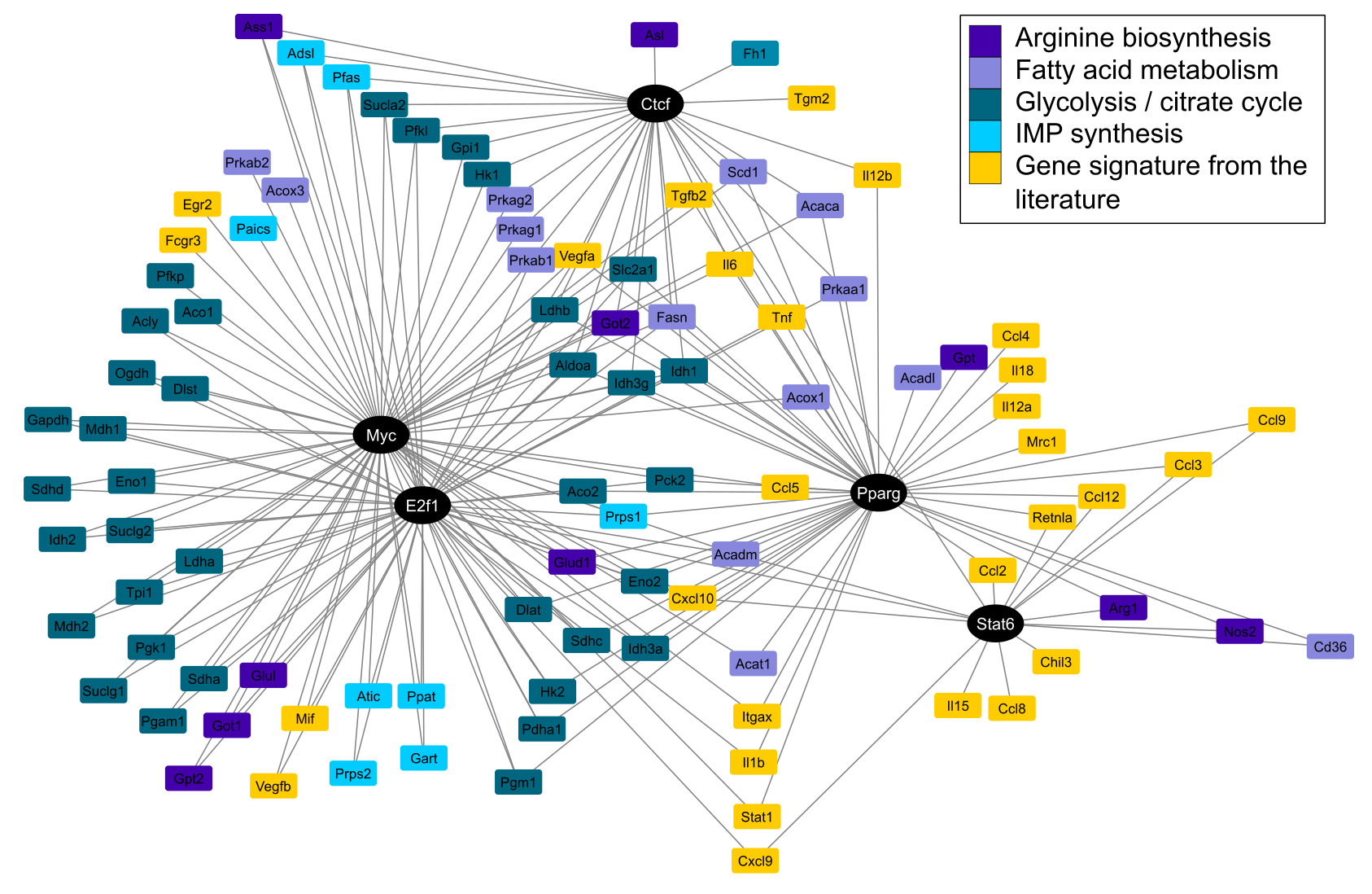

Supplement: S3 Fig — (TIF) [file pcbi.1007657.s003.tif]

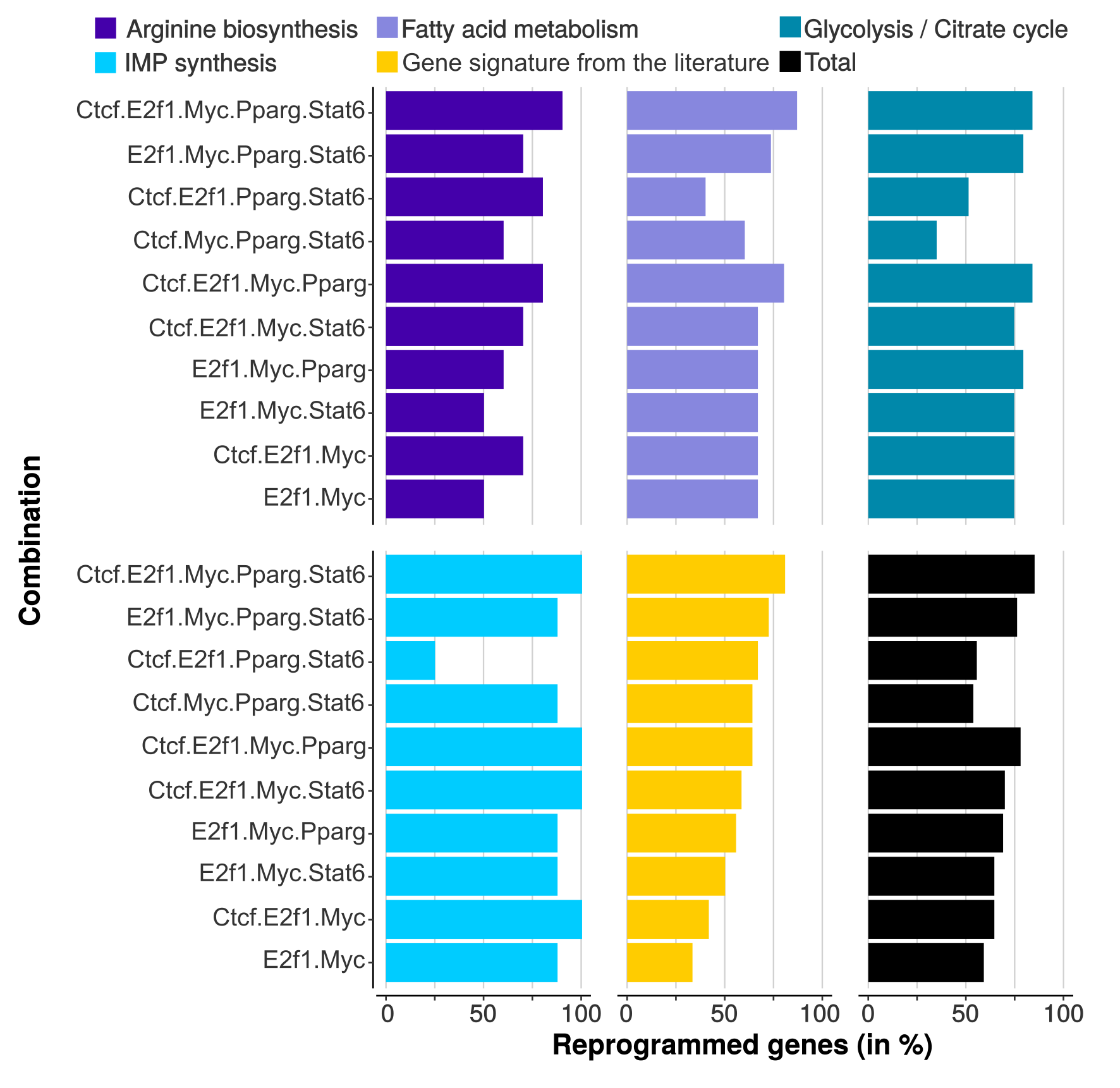

Supplement: S4 Fig — (TIF) [file pcbi.1007657.s004.tif]

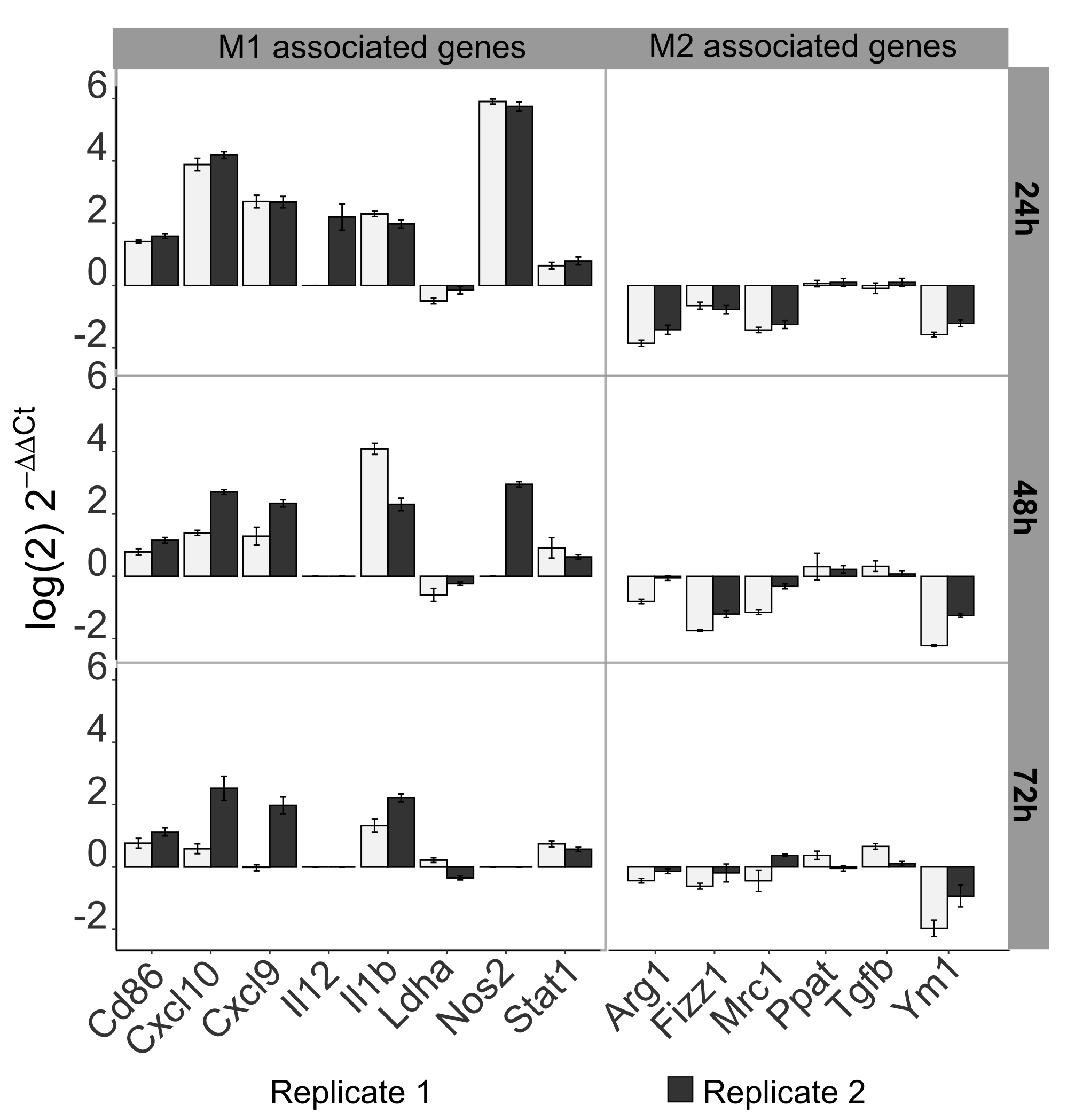

Supplement: S5 Fig — Shown are log2 fold changes in gene expression of 15 genes (from the initial, literature derived signature) in M2 macrophages after treatment with the combined siRNA pool targeting E2f1, Myc, Pparg, Stat6 and Ctcf relative to M2 mock treated macrophages. Samples were extracted 24 h, 48 h and 72 h after siRNA treatment. Error bars are based on the standard error of technical replicates. Gene expression profiles were considered as successfully reprogrammed if a switch towards an M1-like phenotype was observed in both biological replicates (replicate 1 and 2 in grey and black, respectively). (TIF) [file pcbi.1007657.s005.tif]

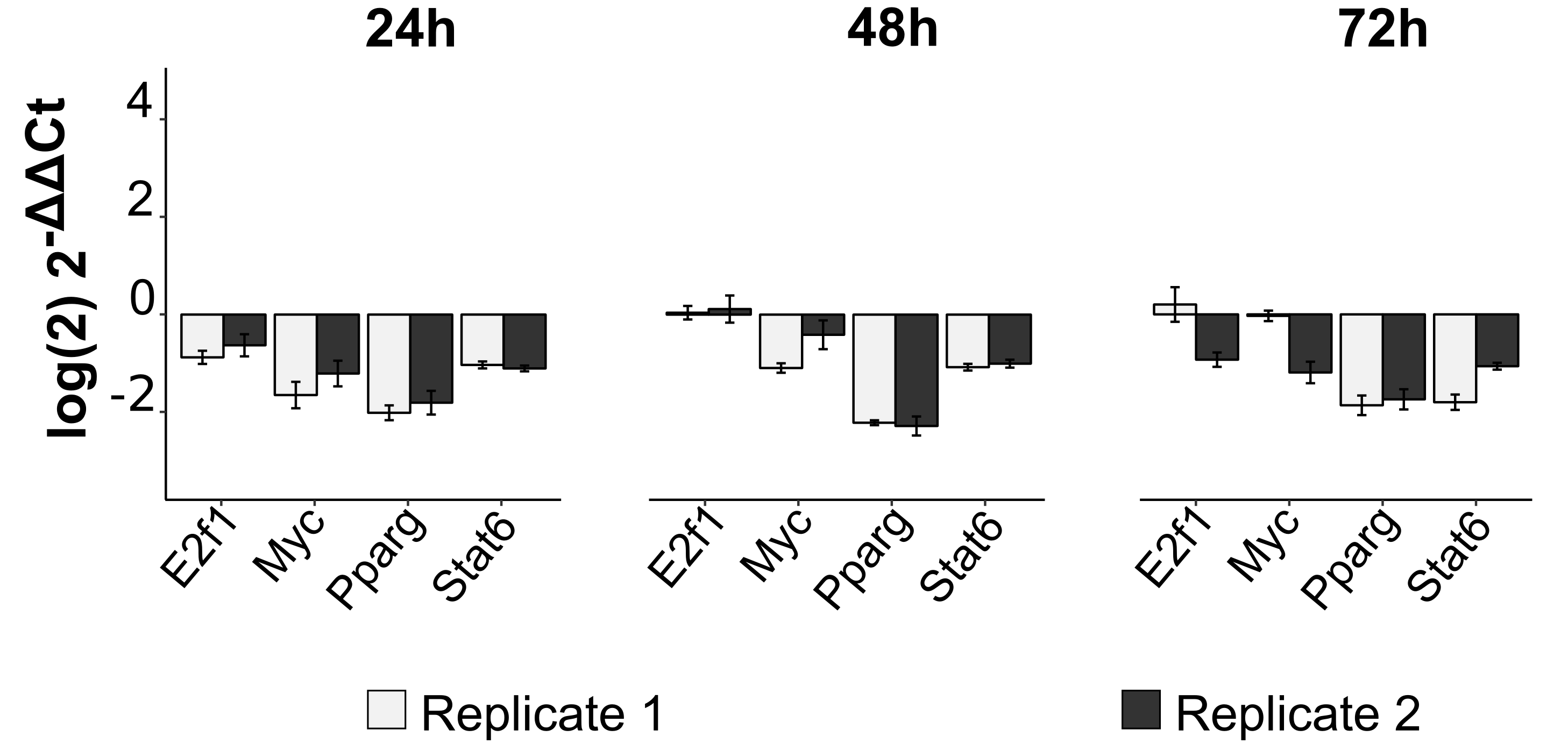

Supplement: S6 Fig — Error bars indicate the standard error of three technical replicates. (TIF) [file pcbi.1007657.s006.tif]

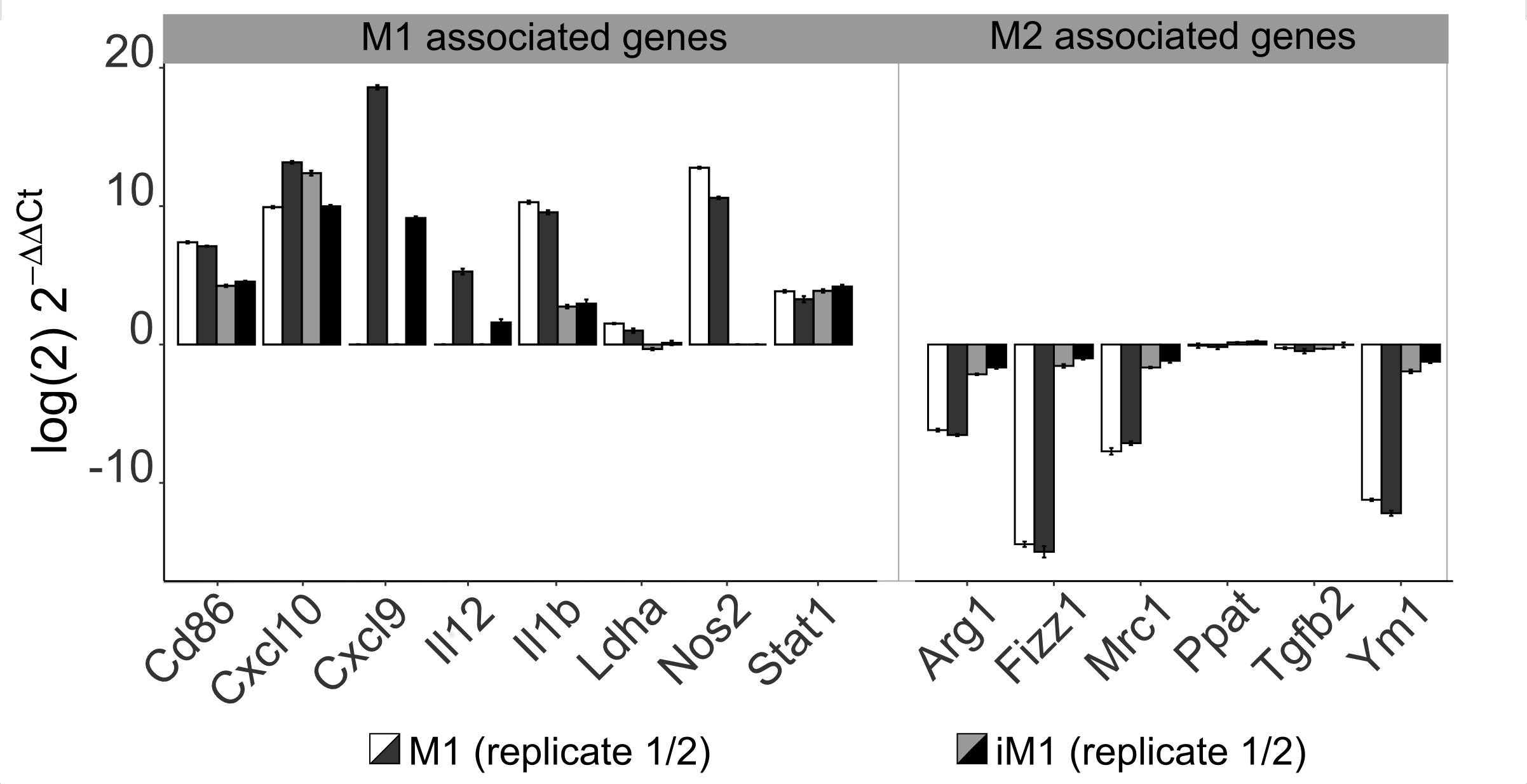

Supplement: S7 Fig — Error bars are based on the standard error of technical replicates. (TIF) [file pcbi.1007657.s007.tif]

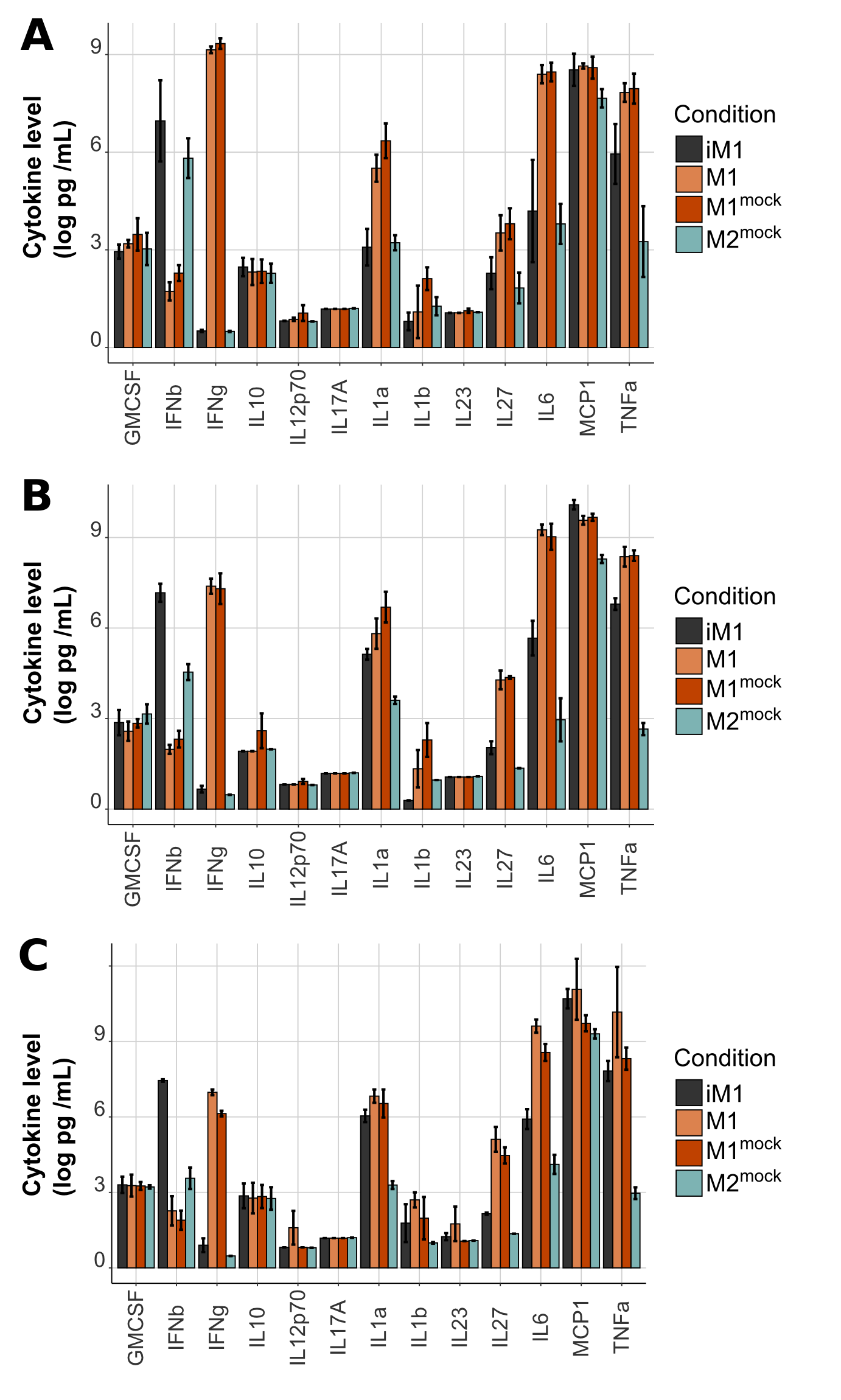

Supplement: S8 Fig — Shown are the concentrations of the cytokines in the medium after 24 hrs (A), 48 hrs (B) and 72 hrs (C), averaged from two technical replicates. (TIF) [file pcbi.1007657.s008.tif]

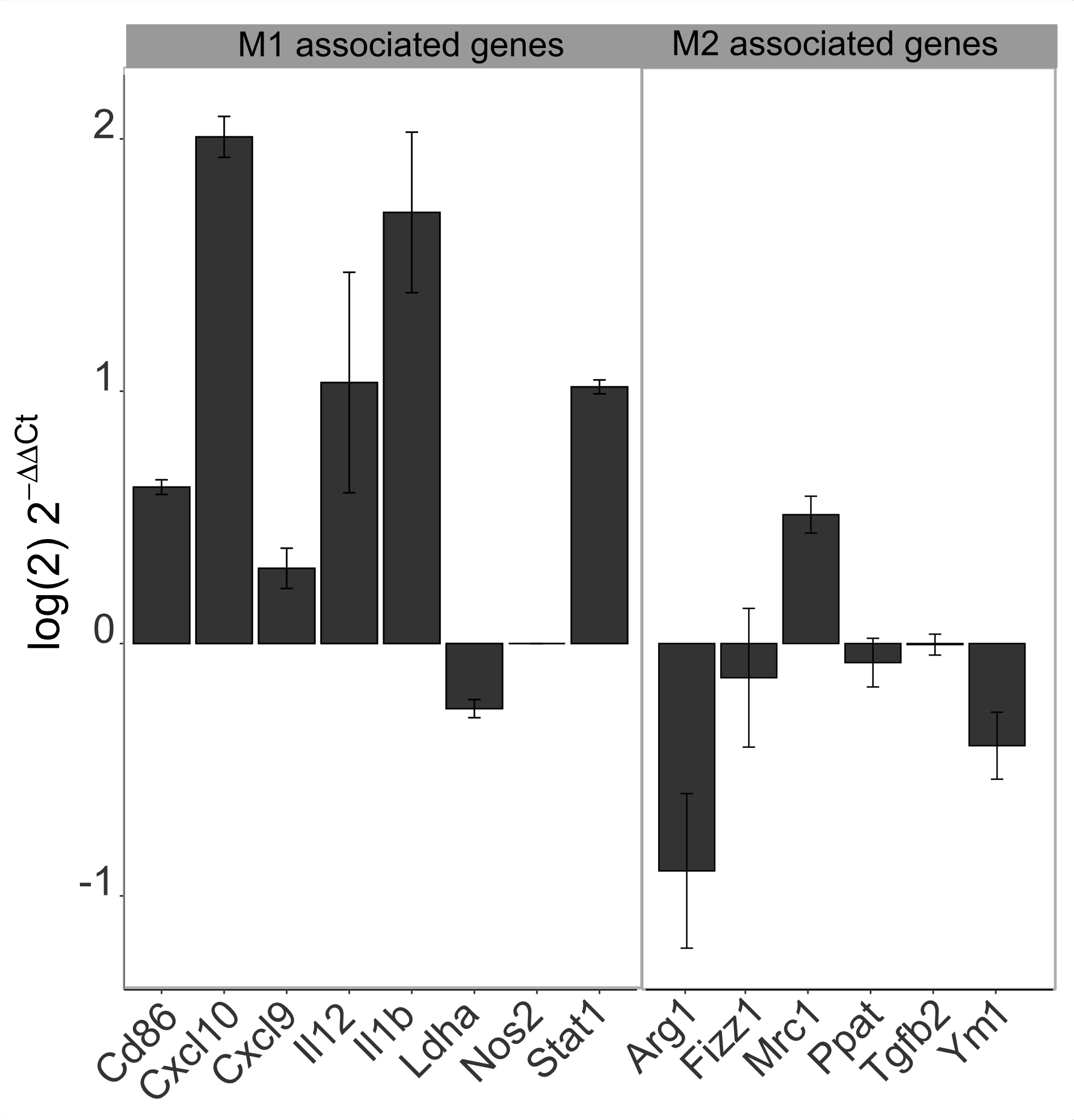

Supplement: S9 Fig — 72 hrs correspond to a similar culturing time of the cells as if we polarize for 24hrs and then transfect with siRNA for 48hrs. Error bars indicate standard deviations of technical replicates. (TIF) [file pcbi.1007657.s009.tif]
