# Supplementary material for: Reprogramming of macrophages employing gene regulatory and metabolic network models
Source: PLoS Comput Biol. 2020 Feb 25;16(2):e1007657. doi: 10.1371/journal.pcbi.1007657 (PMC7059956; doi:10.1371/journal.pcbi.1007657)
Supplement: S4 Table — We assembled a signature from these studies and found very similar differential gene expression (n = 33 agreed, n = 3 disagreed) when comparing expression profiles (M1 versus M2 macrophages) of our experiments to the reported gene expression profiles in literature. (PDF) [file pcbi.1007657.s013.pdf]

Table S4

	Gene symbol	Differential expression of M1 *versus* M2 from our RNA-seq analysis	Reported gene signature	References
M1 associated genes	Ccl2	M1↑	M1↑	[1–3]
	Ccl3	M1↑	M1↑	[1–3]
	Ccl4	M1↑	M1↑	[1–4]
	Ccl5	M1↑	M1↑	[1–4]
	Ccl8	M1↑	M1↑	[2,4]
	Ccl9	M1↑	M1↑	[2]
	Ccl12	M1↑	M1↑	[5]
	Cd80	M1↑	M1↑	[1,2,4]
	Cd86	M1↑	M1↑	[1,2,4]
	Cxcl9	M1↑	M1↑	[1–4,6,7]
	Cxcl10	M1↑	M1↑	[1–4,6]
	Cxcl11	M1↑	M1↑	[1–3,6]
	Il1b	M1↑	M1↑, TAM↑	[1,3,4,7]
	Il6	M1↑	M1↑, TAM↑	[1,3,4,6,7]
	Il12a	M1↑	M1↑	[1–4,6]
	Il12b	M1↑	M1↑	[1–4,6,7]
	Il15	M1↑	M1↑	[8]
	Il18	M1↑	TAM↑	[4]
	Il27	M1↑	M1↑	[6]
	Mif	M1↑	TAM↑	[4]
	Stat1	M1↑	M1↑	[2,6]
	Tnf	M1↑	M1↑	[1–4,6]
M2 associated genes	Chil3 (Ym1)	M2↑	M2↑	[2–4,6]
	Clec10a	M2↑	M2↑, M1↓	[3,7]
	Itgax (Cd11c)	M2↑	M1↑	[9]
	Egr2	M2↑	M1↓	[7]
	Fcgr2	M2↑	M2↑	[3]
	Fcgr3	M2↑	M2↑	[3]
	Mrc1	M2↑	M2↑, M1↓	[3,6,7]
	Retnla (Fizz1)	M2↑	M2↑, M1↓	[1–4,6,7]
	Stab1	M2↑	M2↑	[2,6]
	Stat6	M2↑	M2↑	[3,6]
	Tgfb2	M2↑	M2↑	[2,4,6]
	Tgm2	M1↑	M2↑	[2,6]
	Vegfa	M1↑	M2↑	[2]
	Vegfb	M2↑	CD68+ macrophages↑	[10]

References

1. Mantovani A, Sica A, Sozzani S, Allavena P, Vecchi A, Locati M. The chemokine system in diverse forms of macrophage activation and polarization. Trends in Immunology. 2004. pp. 677–686. doi:10.1016/j.it.2004.09.015

2. Klopfleisch R. Macrophage reaction against biomaterials in the mouse model – Phenotypes, functions and markers. Acta Biomaterialia. 2016. pp. 3–13. doi:10.1016/j.actbio.2016.07.003

3. Gordon S, Martinez FO. The M1 and M2 paradigm of macrophage activation: time for reassessment. F1000Prime Rep. 2014;6: 13.

4. Poh AR, Ernst M. Targeting macrophages in cancer: from bench to bedside. Front Oncol. 2018;8: 49. doi:10.3389/fonc.2018.00049

5. Zhang F, Wang H, Wang X, Jiang G, Liu H, Zhang G, et al. TGF-β induces M2-like macrophage polarization via SNAIL-mediated suppression of a pro-inflammatory phenotype. Oncotarget. 2016;7: 52294–52306. doi:10.18632/oncotarget.10561

6. Murray PJ, Allen JE, Biswas SK, Fisher EA, Gilroy DW, Goerdt S, et al. Macrophage activation and polarization: nomenclature and experimental guidelines. Immunity. 2014. pp. 14–20. doi:10.1016/j.immuni.2014.06.008

7. Jablonski KA, Amici SA, Webb LM, Ruiz-Rosado JDD, Popovich PG, Partida-Sanchez S, et al. Novel markers to delineate murine M1 and M2 macrophages. PLoS One. 2015;10: e0145342. doi:10.1371/journal.pone.0145342

8. Mattiola I, Pesant M, Tentorio PF, Molgora M, Marcenaro E, Lugli E, et al. Priming of human resting NK cells by autologous M1 macrophages via the engagement of IL-1β, IFN-β, and IL-15 pathways. J Immunol. 2015;195: 2818–2828. doi:10.4049/jimmunol.1500325

9. Dempsey WL, Hwu P, Russell DH, Morahan PS. Bone marrow derived macrophages have polyamine and ectoenzyme phenotypes distinct from resident macrophages. Life Sci. 1988;42: 2019–2027. doi:10.1016/0024-3205(88)90502-4

10. Boer K, Troost D, Spliet WGM, Van Rijen PC, Gorter JA, Aronica E. Cellular distribution of vascular endothelial growth factor A (VEGFA) and B (VEGFB) and VEGF receptors 1 and 2 in focal cortical dysplasia type IIB. Acta Neuropathol. 2008;115: 683–696. doi:10.1007/s00401-008-0354-6
