# Supplementary material for: Reprogramming of macrophages employing gene regulatory and metabolic network models
Source: PLoS Comput Biol. 2020 Feb 25;16(2):e1007657. doi: 10.1371/journal.pcbi.1007657 (PMC7059956; doi:10.1371/journal.pcbi.1007657)
Supplement: S5 Table — (PDF) [file pcbi.1007657.s014.pdf]

Table S5

	iM1 versus M2$^{neg\text{-}siPool}$ (qPCR)			log2FC M1 versus M2 (adj. p value < 0.05)**
	24h*	48h	72h	
**Cd86**	2	2	0	5.9
**Cxcl10**	2	2	0	12.7
**Cxcl9**	2	2	0	13.1
**Il12b**	2	1	0	11.3
**Il1b**	2	2	2	9.1
**Ldha**	-1	0	-2	2.4
**Nos2**	2	2	2	13.5
**Stat1**	2	2	0	3.8
**Arg1**	-2	0	0	-1.5
**Fizz1**	-2	-2	-2	-3.5
**Mrc1**	-2	-2	0	-7.3
**Ppat**	2	0	-2	-1.1
**Tgfb**	-1	0	-2	-0.8
**Ym1**	-2	-2	-2	-8.3

* Transcriptional changes of M1 and M2 associated genes relative to M2 macrophages treated with a negative siRNA pool were experimentally determined by quantitative PCR. Samples were extracted 24 h, 48 h and 72 h after siRNA treatment (two replicates). Upregulation or downregulation of a gene was scored for each replicate by 1 or -1, respectively.

** 24 hours after siRNA treatment
