# Supplementary material for: Reprogramming of macrophages employing gene regulatory and metabolic network models
Source: PLoS Comput Biol. 2020 Feb 25;16(2):e1007657. doi: 10.1371/journal.pcbi.1007657 (PMC7059956; doi:10.1371/journal.pcbi.1007657)
Supplement: S6 Table — (PDF) [file pcbi.1007657.s015.pdf]

Table S6

Gene symbol	log2FoldChange* iM1/M2	Adjusted P-value** iM1/M2	log2FoldChange* M1/M2	Adjusted P-value** M1/M2	Correctly reprogrammed (Yes/No)***
Ccl12	2.641	5.20E-10	1.536	3.91E-04	Yes
Ass1****	2.324	2.91E-08	2.080	4.32E-07	Yes
Ccl2	2.218	2.53E-14	2.463	4.52E-18	Yes
Cxcl10	2.178	2.20E-07	0.756	9.46E-02	No
Ccl5	2.093	1.59E-06	4.639	5.81E-30	Yes
Nos2	2.018	2.44E-07	8.796	1.00E-136	Yes
Tnf	1.841	4.05E-21	1.583	2.80E-16	Yes
Ccl4	1.777	2.52E-10	0.214	5.41E-01	No
Ccl8	1.687	6.66E-05	2.560	6.27E-11	Yes
Cd86	1.531	5.09E-29	3.932	2.85E-212	Yes
Il6	1.388	2.63E-03	1.514	4.84E-04	Yes
Cd80	1.134	1.05E-12	1.259	2.65E-16	Yes
Slc2a6	1.130	4.88E-14	0.785	1.76E-07	Yes
Ccl3	1.123	1.23E-19	-0.935	1.24E-13	No
Cxcl9	1.079	2.44E-02	5.861	1.12E-48	Yes
Stat1	0.978	2.20E-18	1.406	2.93E-38	Yes
Mif	0.826	2.35E-03	1.369	1.90E-08	Yes
Il27	0.794	3.08E-02	2.649	5.22E-19	Yes
Tgm2	0.701	5.87E-09	1.952	9.45E-67	Yes
Cxcl11	0.661	1	0.205	5.14E-01	No
Pfkl	0.532	6.17E-06	0.788	1.38E-12	Yes
Il15	0.449	5.93E-03	0.504	8.82E-04	Yes
Slc2a1	0.421	8.98E-06	1.176	5.40E-42	Yes
Arg1	0.411	4.82E-01	0.613	2.09E-01	No
Hk2	0.359	1.33E-06	1.411	2.70E-97	Yes
Adsl	0.304	2.64E-02	0.213	1.11E-01	No
Prkab1	0.296	1.86E-03	0.448	2.23E-07	Yes
Sdhd	0.289	2.44E-03	0.591	5.44E-12	Yes
Eno1	0.283	3.43E-02	1.045	2.80E-21	Yes
Vegfb	0.239	1.25E-01	-0.242	9.08E-02	No
Mdh2	0.185	6.21E-03	0.098	1.54E-01	No
Pfas	0.172	2.75E-01	0.243	6.93E-02	No
Gapdh	0.166	4.19E-01	0.934	9.64E-11	No
Got1	0.163	1.13E-01	-0.017	8.88E-01	No
Stab1	0.137	7.32E-01	-0.779	4.85E-03	No
Dlat	0.129	1.08E-01	0.035	6.86E-01	No
Suclg1	0.127	3.73E-01	0.183	1.28E-01	No
Tpi1	0.120	1.63E-01	0.886	2.09E-39	No
Aco2	0.107	1.92E-01	-0.016	8.58E-01	No
Prkaa1	0.092	4.09E-01	-0.171	6.16E-02	No
Hk1	0.091	4.73E-01	0.707	3.84E-15	No
Bpgm	0.088	6.78E-01	0.555	8.14E-05	No
Dlst	0.078	2.23E-01	0.199	9.77E-05	No

Slc2a9	0.062	8.01E-01	0.592	1.01E-04	No
Pgm1	0.053	6.81E-01	0.117	2.41E-01	No
Got2	0.041	7.60E-01	0.642	1.90E-14	No
Prkag1	0.032	8.80E-01	0.257	6.38E-02	No
Sdhc	0.029	8.61E-01	0.023	8.70E-01	No
Pgk1	0.028	8.65E-01	0.819	2.57E-18	No
Prps1	0.022	9.34E-01	0.330	4.49E-02	No
Il1b	0.007	9.94E-01	0.764	1.06E-01	No
Il12b	0.000	1	1.225	3.02E-04	No
Asl	-0.028	8.35E-01	-0.464	3.34E-08	No
Mdh1	-0.059	6.90E-01	0.066	6.01E-01	No
Acox3	-0.059	5.72E-01	-0.183	1.92E-02	No
Idh3a	-0.077	5.88E-01	0.067	5.86E-01	No
Pdha1	-0.089	2.74E-01	0.181	5.38E-03	No
Ccl9	-0.089	8.06E-01	-5.613	1.82E-132	No
Idh3g	-0.096	4.63E-01	-0.283	4.99E-03	No
Il12a	-0.096	1	0.517	1	No
Gart	-0.096	4.25E-01	-0.267	4.52E-03	No
Sdha	-0.110	7.50E-02	-0.299	1.24E-08	No
Fh1	-0.120	4.07E-01	-0.444	5.93E-05	No
Gpi1	-0.134	2.61E-01	0.131	2.21E-01	No
Pgm2	-0.138	1.99E-01	0.008	9.45E-01	No
Sucla2	-0.142	1.46E-01	-0.223	8.06E-03	No
Gpt	-0.143	5.82E-01	0.017	9.48E-01	No
Fcgr2b	-0.154	3.69E-01	-1.594	2.09E-36	No
Acat1	-0.161	2.11E-01	-0.097	4.29E-01	No
Ldha	-0.162	1.44E-01	0.730	1.16E-16	No
Prkab2	-0.171	3.97E-01	0.194	2.54E-01	No
Il18	-0.190	4.85E-01	-0.096	7.05E-01	No
Pfkp	-0.214	1.08E-01	0.502	7.33E-06	No
Idh2	-0.215	5.00E-02	-0.564	2.82E-09	Yes
Hk3	-0.218	5.24E-04	0.613	3.42E-27	No
Ogdh	-0.222	3.66E-03	-0.836	1.17E-34	Yes
Mrc1	-0.226	2.09E-01	-3.662	7.34E-147	No
Acox1	-0.238	3.65E-04	-0.130	5.26E-02	No
Acadl	-0.258	2.63E-03	-0.359	5.78E-06	Yes
Ppat	-0.261	3.88E-02	-0.239	4.15E-02	Yes
Pgam1	-0.266	4.50E-03	0.366	1.56E-05	No
Acadm	-0.273	9.66E-05	-0.409	4.29E-10	Yes
Egr2	-0.282	3.81E-02	-2.801	2.14E-123	Yes
Aco1	-0.286	1.84E-04	-0.703	1.89E-23	Yes
Prkag2	-0.297	1.88E-02	-0.095	4.83E-01	No
Suclg2	-0.325	2.45E-01	-0.267	2.92E-01	No
Atic	-0.347	4.18E-06	-0.616	5.26E-18	Yes
Vegfa	-0.371	1.34E-01	1.446	5.90E-13	No
Aldoa	-0.383	4.38E-05	-0.308	7.90E-04	Yes
Fcgr3	-0.386	1.25E-04	-0.151	1.57E-01	No

Gpt2	-0.394	4.05E-03	-0.339	9.16E-03	Yes
Glud1	-0.412	1.02E-14	-0.778	1.69E-51	Yes
Acly	-0.450	8.20E-11	-0.519	1.14E-14	Yes
Pck2	-0.476	2.29E-01	-0.601	8.26E-02	No
Itgax	-0.495	1.38E-02	-4.880	1.10E-174	Yes
Slc2a8	-0.509	1.43E-04	-0.004	9.82E-01	No
Paics	-0.532	4.54E-17	-0.318	3.64E-07	Yes
Eno2	-0.603	1.88E-05	-1.696	1.76E-37	Yes
Glul	-0.661	9.66E-34	-1.304	5.50E-126	Yes
Chil3	-0.702	5.20E-04	-6.943	4.40E-287	Yes
Cd36	-0.730	4.64E-08	-3.206	3.37E-145	Yes
Idh1	-0.732	1.58E-19	-2.158	3.60E-162	Yes
Ldhb	-0.742	1.81E-04	-0.288	1.60E-01	No
Prps2	-0.753	1.74E-06	0.206	2.19E-01	No
Stat6	-0.863	6.11E-20	-0.045	7.13E-01	No
Acaca	-1.021	8.10E-25	-1.305	2.07E-40	Yes
Retnla	-1.085	1.82E-02	-4.231	1.34E-26	Yes
Clec10a	-1.613	1.35E-12	-1.772	1.46E-15	Yes
Fasn	-1.804	9.88E-26	-3.268	4.61E-81	Yes
Tgfb2	-1.941	4.69E-16	-2.003	7.46E-18	Yes
Scd1	-2.977	6.70E-43	-5.340	5.25E-113	Yes
Scd2	-3.117	9.94E-38	-3.308	2.13E-43	Yes

* DESeq2 was used for differential expression analysis. The reported result comprises the gene signature between iM1 and M2-like (mock treated) macrophages and M1 (mock treated) and M2-like (mock treated) macrophages.

** The Benjamini-Hochberg method was used for multiple testing correction

*** Compared to the differential expression of the M1-like and M2-like macrophages not treated with the transfection reagent

**** Enzyme coding genes are highlighted in gray
