# Supplementary material for: Reprogramming of macrophages employing gene regulatory and metabolic network models
Source: PLoS Comput Biol. 2020 Feb 25;16(2):e1007657. doi: 10.1371/journal.pcbi.1007657 (PMC7059956; doi:10.1371/journal.pcbi.1007657)
Supplement: S7 Table — (DOCX) [file pcbi.1007657.s016.docx]

**Table S7.** Primers for quantitative real-time PCR. Primers were purchased from Sigma-Aldrich (St. Louis, USA), resolved in ddH_2_O to a stock concentration of 100 µM and stored at -20 °C

| **Primer** | **Sequence (5'-3')** | **Source** |
| --- | --- | --- |
| Ym1_qPCR_FP1 | CACCATGGCCAAGCTCATTCTTGT | Tatano *et al.* 2014[1] |
| Ym1_qPCR_RP2 | TATTGGCCTGTCCTTAGCCCAACT |  |
| Fizz1_qPCR_FP3 | ACTGCCTGTGCTTACTCGTTGACT |  |
| Fizz1_qPCR_RP4 | AAAGCTGGGTTCTCCACCTCTTCA |  |
| CXCL10_qPCR_FP7 | TCTGAGTCCTCGCTCAAGTG | Movahedi *et al.* |
| CXCL10_qPCR_RP8 | CCTTGGGAAGATGGTGGTTA | 2010[2] |
| IL1b_qPCR_FP13 | GTGTGGATCCAAAGCAATAC |  |
| IL1b_qPCR_RP14 | GTCTGCTCATTCATGACAAG |  |
| ARG1_qPCR_FP19 | TCACCTGAGCTTTGATGTCG |  |
| ARG1_qPCR_RP20 | TTATGGTTACCCTCCCGTTG |  |
| CD206_qPCR_FP23 | TTGGACGGATAGATGGAGGG | Zhu *et al.* |
| CD206_qPCR_RP24 | CCAGGCAGTTGAGGAGGTTC | 2014[3] |
| STAT6_fw37 | CTGGGGTGGTTTCCTCTTG | Shaul *et al.* |
| STAT6_rev38 | TGCCCGGTCTCACCTAACTA | 2010[4] |
| IL1β_fw39 | CTGGTGTGTGACGTTCCCATTA |  |
| IL1β_rev40 | CCGACAGCACGAGGCTTT |  |
| STAT1_fw41 | CTGAATATTTCCCTCCTGGG |  |
| STAT1_rev42 | TCCCGTACAGATGTCCATGAT |  |
| CD86_fw45 | TCTCCACGGAAACAGCATCT |  |
| CD86_rev46 | CTTACGGAAGCACCCATGAT |  |
| TGFβ1_fw49 | AAGTTGGCATGGTAGCCCTT |  |
| TGFβ1_rev50 | GCCCTGGATACCAACTATTGC |  |
| IL12b_fw57 | AGTGACATGTGGAATGGCGT | Designed by  David Eisel |
| IL12b_rev58 | CAGGAGTCAGGGTACTCCCA |  |
| Rpl19_fw69 | TACCGGGAATCCAAGAAGATTGA | PrimerBank ID [5–7] 226958656c3 |
| Rpl19_rev70 | AGGATGCGCTTGTTTTTGAAC |  |
| Ldha_fw71 | TGTCTCCAGCAAAGACTACTGT | PrimerBank ID 6754524a1 |
| Ldha_rev72 | GACTGTACTTGACAATGTTGGGA |  |
| Ppat_fw75 | TTCAGGGTGCATAAGGGAATGG | PrimerBank ID 247301190c1 |
| Ppat_rev76 | GCGTACCTCGTATGTCCGA |  |
| Nos2_fw81 | GTTCTCAGCCCAACAATACAAGA | PrimerBank ID  6754872a1 |
| Nos2_rev82 | GTGGACGGGTCGATGTCAC |  |
| CXCL9_fw83 | GGAGTTCGAGGAACCCTAGTG | PrimerBank ID 162287427c1 |
| CXCL9_rev84 | GGGATTTGTAGTGGATCGTGC |  |
| Myc_fw89 | ATGCCCCTCAACGTGAACTTC | PrimerBank ID 293629266c1 |
| Myc_rev90 | GTCGCAGATGAAATAGGGCTG |  |
| Pparg_fw99 | TTTTCCGAAGAACCATCCGATT | PrimerBank ID 187960104c3 |
| Pparg_rev100 | ATGGCATTGTGAGACATCCCC |  |
| Stat6_fw103 | TGGAGAGCATCTATCAGAGGGA | PrimerBank ID 128485773c3 |
| Stat6_rev104 | GCGGAACTCTTCTATAACAGCTT |  |
| Ctcf_fw125 | GATCCTACCCTTCTCCAGATGAA | PrimerBank ID 31044459a1 |
| Ctcf_rev126 | GTACCGTCACAGGAACAGGT |  |
| E2f1_fw127 | TGCAGAAACGGCGCATCTAT | PrimerBank ID 158517881c2 |
| E2f1_rev128 | CCGCTTACCAATCCCCACC |  |

**References**

1. Tatano Y, Shimizu T, Tomioka H. Unique macrophages different from M1/M2 macrophages inhibit T cell mitogenesis while upregulating Th17 polarization. Sci Rep. 2015;4: 4146. doi:10.1038/srep04146

2. Movahedi K, Laoui D, Gysemans C, Baeten M, Stange G, Van den Bossche J, et al. Different tumor microenvironments contain functionally distinct subsets of macrophages derived from Ly6C(high) monocytes. Cancer Res. 2010;70: 5728–5739. doi:10.1158/0008-5472.CAN-09-4672

3. Zhu L, Yang T, Li L, Sun L, Hou Y, Hu X, et al. TSC1 controls macrophage polarization to prevent inflammatory disease. Nat Commun. 2014;5: 4696. doi:10.1038/ncomms5696

4. Shaul ME, Bennett G, Strissel KJ, Greenberg AS, Obin MS. Dynamic, M2-like remodeling phenotypes of CD11c+ adipose tissue macrophages during high-fat diet-induced obesity in mice. Diabetes. 2010;59: 1171–1181. doi:10.2337/db09-1402

5. Spandidos A, Wang X, Wang H, Dragnev S, Thurber T, Seed B. A comprehensive collection of experimentally validated primers for Polymerase Chain Reaction quantitation of murine transcript abundance. BMC Genomics. 2008;9: 633. doi:10.1186/1471-2164-9-633

6. Spandidos A, Wang X, Wang H, Seed B. PrimerBank: a resource of human and mouse PCR primer pairs for gene expression detection and quantification. Nucleic Acids Res. 2010;38: D792–D799. doi:10.1093/nar/gkp1005

7. Wang X, Seed B. A PCR primer bank for quantitative gene expression analysis. Nucleic Acids Res. 2003;31: e154. Available: http://www.ncbi.nlm.nih.gov/pubmed/14654707
